# Body Mass Index and Risk of Colorectal Cancer Incidence and Mortality in Asia

**DOI:** 10.1001/jamanetworkopen.2024.29494

**Published:** 2024-08-28

**Authors:** Pedram Paragomi, Zhongjie Zhang, Sarah Krull Abe, Md. Rashedul Islam, Md. Shafiur Rahman, Eiko Saito, Xiao-Ou Shu, Bashir Dabo, Yen Thi-Hai Pham, Yu Chen, Yu-Tang Gao, Woon-Puay Koh, Norie Sawada, Reza Malekzadeh, Ritsu Sakata, Atsushi Hozawa, Jeongseon Kim, Seiki Kanemura, Chisato Nagata, San-Lin You, Hidemi Ito, Sue K. Park, Jian-Min Yuan, Wen-Harn Pan, Wanqing Wen, Renwei Wang, Hui Cai, Shoichiro Tsugane, Akram Pourshams, Yumi Sugawara, Keiko Wada, Chien-Jen Chen, Isao Oze, Aesun Shin, Habibul Ahsan, Paolo Boffetta, Kee Seng Chia, Keitaro Matsuo, You-Lin Qiao, Nathaniel Rothman, Wei Zheng, Manami Inoue, Daehee Kang, Hung N. Luu

**Affiliations:** 1 Division of Cancer Control and Population Sciences, University of Pittsburgh Medical Center Hillman Cancer Center, University of Pittsburgh Medical Center, Pittsburgh, Pennsylvania; 2 Department of Epidemiology, University of Pittsburgh School of Public Health, Pittsburgh, Pennsylvania; 3 Division of Prevention, National Cancer Center Institute for Cancer Control, Tokyo, Japan; 4 Department of Global Health Policy, Graduate School of Medicine, The University of Tokyo, Tokyo, Japan; 5 Research Center for Child Mental Development, Hamamatsu University School of Medicine, Hamamatsu, Japan; 6 Institute for Global Health Policy Research, National Center for Global Health and Medicine, Tokyo, Japan; 7 Division of Epidemiology, Department of Medicine, Vanderbilt Epidemiology Center, Vanderbilt-Ingram Cancer Center, Vanderbilt University Medical Center, Nashville, Tennessee; 8 Department of Epidemiology and Biostatistics, College of Public Health, University of South Florida, Tampa; 9 Department of Population Health, New York University Grossman School of Medicine, New York; 10 Division of Environmental Medicine, New York University Grossman School of Medicine, New York; 11 Department of Epidemiology, Shanghai Cancer Institute, Shanghai, China; 12 Renji Hospital, Shanghai Jiaotong University School of Medicine, Shanghai, China; 13 Healthy Longevity Translational Research Programme, Yong Loo Lin School of Medicine, National University of Singapore, Singapore; 14 Singapore Institute for Clinical Sciences, Agency for Science Technology and Research, Singapore; 15 Division of Cohort Research, National Cancer Center Institute for Cancer Control, Tokyo, Japan; 16 Digestive Oncology Research Center, Digestive Diseases Research Institute, Tehran University of Medical Sciences, Tehran, Iran; 17 Radiation Effects Research Foundation, Hiroshima, Japan; 18 Tohoku University Graduate School of Medicine, Sendai, Japan; 19 Graduate School of Science and Policy, National Cancer Center, Gyeonggi-Do, Republic of Korea; 20 Department of Epidemiology and Preventive Medicine, Gifu University Graduate School of Medicine, Gifu, Japan; 21 School of Medicine and Big Data Research Center, Fu Jen Catholic University, New Taipei City, Taiwan; 22 Division of Cancer Information and Control, Department of Preventive Medicine, Aichi Cancer Center Research Institute, Nagoya, Japan; 23 Department of Preventive Medicine, Seoul National University College of Medicine, Seoul, Republic of Korea; 24 Institute of Biomedical Sciences, Academia Sinica, Taipei, Taiwan; 25 Institute of Population Health Sciences, National Health Research Institutes, Miaoli County, Taiwan; 26 Genomics Research Center, Academia Sinica, Taipei, Taiwan; 27 Division of Cancer Epidemiology and Prevention, Aichi Cancer Center Research Institute, Nagoya, Japan; 28 Cancer Research Institute, Seoul National University, Seoul, Republic of Korea; 29 Department of Public Health Sciences, The University of Chicago, Chicago, Illinois; 30 Stony Brook Cancer Center, Stony Brook University, Stony Brook, New York; 31 Department of Medical and Surgical Sciences, University of Bologna, Bologna, Italy; 32 Saw Swee Hock School of Public Health, National University of Singapore, Singapore; 33 Department of Cancer Epidemiology, Nagoya University Graduate School of Medicine, Nagoya, Japan; 34 Center for Global Health, School of Population Medicine and Public Health, Chinese Academy of Medical Sciences and Peking Union Medical College, Beijing, China; 35 Division of Cancer Epidemiology and Genetics, Occupational and Environmental Epidemiology Branch, National Cancer Institute, Bethesda, Maryland

## Abstract

**Importance:**

The global burden of obesity is increasing, as are colorectal cancer (CRC) incidence and mortality.

**Objectives:**

To assess the association between body mass index (BMI) and risks of incident CRC and CRC-related death in the Asian population.

**Design, Setting, and Participants:**

This cohort study includes data pooled from 17 prospective cohort studies included in The Asia Cohort Consortium. Cohort enrollment was conducted from January 1, 1984, to December 31, 2002. Median follow-up time was 15.2 years (IQR, 12.1-19.2 years). Data were analyzed from January 15, 2023, through January 15, 2024.

**Exposure:**

Body mass index, calculated as weight in kilograms divided by height in meters squared.

**Main Outcomes and Measures:**

The primary outcomes were CRC incidence and CRC-related mortality. The risk of events is reported as adjusted hazard ratios (AHRs) and 95% CIs for incident CRC and death from CRC using the Cox proportional hazards regression model.

**Results:**

To assess the risk of incident CRC, 619 981 participants (mean [SD] age, 53.8 [10.1] years; 52.0% female; 11 900 diagnosed incident CRC cases) were included in the study, and to assess CRC-related mortality, 650 195 participants (mean [SD] age, 53.5 [10.2] years; 51.9% female; 4550 identified CRC deaths) were included in the study. A positive association between BMI and risk of CRC was observed among participants with a BMI greater than 25.0 to 27.5 (AHR, 1.09 [95% CI, 1.03-1.16]), greater than 27.5 to 30.0 (AHR, 1.19 [95% CI, 1.11-1.29]), and greater than 30.0 (AHR, 1.32 [95% CI, 1.19-1.46]) compared with those with a BMI greater than 23.0 to 25.0 (*P* < .001 for trend), and BMI was associated with a greater increase in risk for colon cancer than for rectal cancer. A similar association between BMI and CRC-related death risk was observed among participants with a BMI greater than 27.5 (BMI >27.5-30.0: AHR, 1.18 [95% CI, 1.04-1.34]; BMI >30.0: AHR, 1.38 [95% CI, 1.18-1.62]; *P* < .001 for trend) and was present among men with a BMI greater than 30.0 (AHR, 1.87 [95% CI, 1.49-2.34]; *P* < .001 for trend) but not among women (*P* = .15 for trend) (*P* = .02 for heterogeneity).

**Conclusions and Relevance:**

In this cohort study that included a pooled analysis of 17 cohort studies comprising participants across Asia, a positive association between BMI and CRC incidence and related mortality was found. The risk was greater among men and participants with colon cancer. These findings may have implications to better understand the burden of obesity on CRC incidence and related deaths in the Asian population.

## Key Points

**Question:**

What is the association between body mass index (BMI) and colorectal cancer (CRC) incidence and mortality in the Asian population?

**Findings:**

This cohort study from The Asia Cohort Consortium that comprises data from multiple countries across Asia demonstrated positive associations between BMI and risk of incident CRC among 619 981 participants and between BMI and risk of death from CRC among 650 195 participants. Risk was greater among participants with colon cancer compared with rectal cancer and among men compared with women.

**Meaning:**

The findings of this study suggest a positive association between BMI and risks of incident CRC and related mortality in the Asian population, which may offer greater understanding of the burden of obesity on CRC incidence and related deaths in this population.

## Teaser

This cohort study assesses whether body mass index is associated with colorectal cancer incidence and mortality in Asian populations.

## Introduction

Colorectal cancer (CRC) is one of the leading causes of morbidity and mortality in the US and Europe and many other parts of the world.^1^ Colorectal cancer constitutes 10% of global cancer incidence and about 9.4% of total cancer-related mortality.^2^ The annual incidence of CRC was estimated at 1.9 million cases in 2020, and it is projected that annual incident CRC cases will reach 3.2 million by 2040.^3^ The general consensus is that CRC is a marker of socioeconomic development with incidence rates reflecting the human development index.^4^ The surge of CRC is mainly attributed to changes in lifestyle, environmental exposures, and dietary risk factors.^5^ In many Asian countries, the incidence of CRC has been rising, following the increased trend in Western countries.^6^

The global prevalence of obesity has shown an upward trajectory between 1975 and 2016.^7,8^ The adoption of a Westernized lifestyle may contribute to a high prevalence of obesity in Asian countries. For instance, in 2020, 34.3% of Chinese adults were categorized as overweight and 16.4% as obese according to the World Health Organization’s definitions.^9^ Similarly, data from other Asian countries have indicated a steady increase in the prevalence of overweight and obesity in accordance with economic growth and changes in dietary habits.^10^

The association between body mass index (BMI) and incidence of CRC has been evaluated in Asia, including China,^11^ Japan,^12,13^ Korea,^14,15^ Singapore,^16^ and Taiwan.^17^ Accordingly, a pooled analysis of more than 300 000 Japanese individuals reported an increased risk of CRC incidence among study participants who were overweight or obese.^18^ Results of a prospective cohort study of more than 61 000 individuals from Singapore showed a U-shaped association between BMI and incidence of colon cancer or an increased risk of CRC in individuals who were both underweight (BMI <18.5) and overweight or obese (BMI ≥27.5) compared with individuals with what is considered a normal BMI (between 18.5 and 23.0)^16^ (calculated as weight in kilograms divided by height in meters squared). The Asian Pacific Working Group on Colorectal Cancer consensus recommendations showed that obesity is a risk factor for CRC development, among other factors such as being male, having a family history of cancer, and smoking.^19^

Efforts have been made to determine the association between BMI and risk of CRC mortality in the US and Australia.^20,21^ For instance, the Cancer Prevention Study II, a prospective mortality study of 1 184 659 US adults, reported that BMI was associated with colon cancer death in men.^22^ The association between BMI and CRC mortality, however, has been relatively understudied in Asia. For example, the Asia-Pacific Cohort Studies Collaboration, which included only 668 CRC deaths, found an increased risk of CRC mortality among individuals with obesity.^23^ The Japan Collaborative Cohort Study for Evaluation of Cancer, which included 127 colon cancer deaths, reported a positive association between BMI and cancer-related mortality among women only.^24^ There is a gap of knowledge on the association between BMI and CRC incidence and related mortality across Asia.

In the current study, we examined comprehensively the association between BMI and risk of CRC incidence and mortality across Asia. We included pooled data from The Asia Cohort Consortium that included more than 1 million participants.

## Methods

### Study Populations

Data for this cohort study were derived from The Asia Cohort Consortium (ACC), an international collaborative effort including more than 1 million participants across Asia. Details of the ACC have been described in previous studies.^25,26,27^ All participants were required to sign an informed consent form. Each cohort received approval from its respective institutional review board to conduct research, and the pooled analysis was approved by the ACC Executive Committee and by the Ethical Committee of the National Cancer Center Japan (additional details are provided in the eMethods in Supplement 1). This study followed the Strengthening the Reporting of Observational Studies in Epidemiology (STROBE) reporting guideline.

In this study, a total of 17 cohorts from mainland China, Japan, South Korea, Taiwan, Singapore, and Iran were included. Cohort enrollment was conducted from January 1, 1984, to December 31, 2002. Median follow-up time was 15.2 years (IQR, 12.1-19.2 years). Due to the limited data on the date of CRC diagnosis, 2 cohorts from Taiwan were excluded from CRC incidence analysis, while the CRC mortality analysis was performed using all cohorts.

For analysis of CRC incidence, the exclusion criteria included (1) a missing or an extreme BMI value (>80 or <10) at baseline, (2) the unknown status of CRC diagnosis during the follow-up period, (3) unavailable time to CRC diagnosis, (4) CRC not being the first primary cancer diagnosed, or (5) missing data on potential confounding factors. To prevent reverse causality, we also excluded participants with CRC and person-years observed within the first 3 years of observation after the enrollment.

Similarly, the following exclusion criteria were used in the analysis of CRC mortality: (1) a missing or an extreme BMI value (>80 or <10) at baseline; (2) missing vital status; (3) missing the date of CRC death; and (4) missing data on potential confounding factors, including age at baseline, diabetes, being a current smoker, and ever using alcohol. Deaths from CRC and person-years observed within the first 3 years of observation after the enrollment were excluded to minimize reverse causality.

### Primary Outcomes, Exposures, and Other Covariates

Data on CRC incidence and related mortality were provided by each participating cohort, in which cancer diagnosis was established through linkage to local cancer registries. Colon cancer was identified as *International Classification of Diseases, Ninth Revision* (*ICD-9*) code 153 and *International Statistical Classification of Diseases and Related Health Problems, Tenth Revision* (*ICD-10*) codes C18 and D37.4. Rectal cancer included *ICD-9* codes 154, 154.0, 154.1, 154.4, 154.8, and 154.9 and *ICD-10* codes C19, C20, and D37.5. For those who had a CRC diagnosis with a missing *ICD-9* or *ICD-10* code, we included those participants in the incidence analysis, but they were excluded from the subsite analyses. We constructed the time to event by computing the interval between the date of enrollment and the date of incident CRC or death from CRC. For participants not experiencing these 2 outcomes, they were censored at the date of last contact or the date of death from other causes, whichever came first.

Body mass index was obtained from either the measurement provided by cohorts (11 cohorts) or calculated using self-reported height and weight (6 cohorts). Consistent with a previous analysis in the ACC,^28^ we used the following cutoffs: less than 18.5, 18.5 to 23.0, greater than 23.0 to 25.0, greater than 25.0 to 27.5, greater than 27.5 to 30.0, greater than 30.0 to 35.0, and greater than 35.0, in which greater than 23.0 to 25.0 was considered the reference group. A previous study in the ACC revealed that the BMI category of 23.0 to 25.0 had the lowest risk of all-cause mortality.^28^ As there were few CRC cases or related deaths in the category of greater than 35.0 (<1% in all cohorts except the Golestan [Iran] cohort), we combined this category with the greater than 30.0 to 35.0 category.

### Statistical Analysis

Data were analyzed from January 15, 2023, to January 15, 2024. Adjusted hazard ratios (AHRs) and 95% CIs were calculated using Cox proportional hazards regression models, in which the cohort indicator was included as a random effect term to account for between-study heterogeneity. For primary analyses, multivariable AHRs were obtained after adjusting for age, sex, diabetes, current smoking status, and ever alcohol use. A further stratified analysis was performed by sex, country, diabetes, smoking status, and alcohol use. We examined the statistical significance of the product term between BMI and the corresponding stratification factor in multivariable models to assess whether heterogeneity existed in any of these subgroups. Potential sex difference was also explored in each subgroup of country, diabetes, smoking, and alcohol use. The definition of a complete dataset with covariates included those with complete information of age at baseline, sex, educational level, marital status, diabetes at baseline, current smoker, ever alcohol use, and enrollment period.

Linear trends between BMI with CRC incidence and mortality were assessed by including a continuous variable that took the median BMI of each BMI subgroup using Wald tests. To assess the nonlinear associations between BMI and CRC incidence and mortality, a restricted cubic spline analysis was performed in which we set a BMI of 24.0 as the reference value, and 4 knots were placed at equally spaced percentiles given the distribution of BMI in the specific study population as previously recommended.^29^

All statistical analyses were performed using SAS, version 9.4 (SAS Institute Inc) or Stata, version 17.0 (StataCorp LLC). All *P* values were 2-sided, and *P* < .05 was the threshold for statistical significance. Bonferroni correction was used for subgroup analyses.

## Results

### Pooled Data

There were a total of 709 214 individuals included from 17 cohorts. With a median follow-up of 15.2 years (IQR, 12.1-19.2 years), 11 900 incident CRC cases were diagnosed among 619 981 participants from 15 ACC participating cohorts (mean [SD] age, 53.8 [10.1] years; 52.0% female; 48.0% male), and 4 550 CRC-related deaths were identified from all 17 ACC cohorts that included 650 195 participants (mean [SD] age, 53.5 [10.2] years; 51.9% female; 48.1% male). The overall mean (SD) for BMI was 23.4 (3.2), and in the population, 66.3% had a BMI between 18.5 and 25.0, 25.4% were obese (BMI between >25.0 and 30.0), and 3.5% had a BMI greater than 30.0 (eTables 1 and 2 in Supplement 1).

### BMI and Risk of CRC Incidence

**Figure 1.**
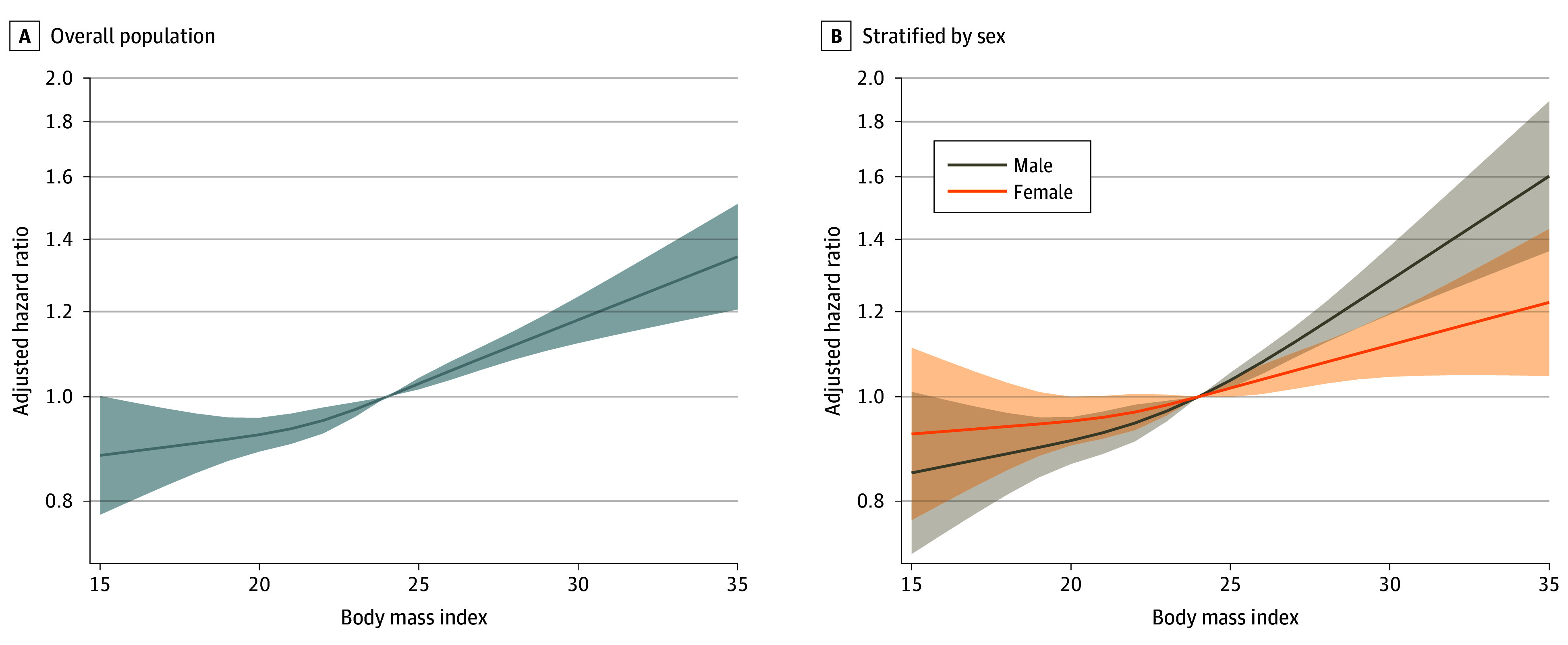
Association Between Body Mass Index and Risk of Incident Colorectal Cancer A, Model adjusted for age at baseline, sex, diabetes at baseline, current smoker, and ever alcohol use. B, Model adjusted for age at baseline, diabetes at baseline, current smoker, and ever alcohol use. Body mass index was calculated as weight in kilograms divided by height in meters squared. Shaded areas indicate 95% CIs.

We observed an increased risk of CRC incidence in individuals with a BMI greater than 25.0 compared with individuals with what is considered normal BMI (ie, >23.0-25.0). Specifically, the risk of developing CRC consistently increased from 9% to 32% in individuals with a BMI greater than 25.0 (>25.0-27.5: AHR, 1.09 [95% CI, 1.03-1.16]; >27.5-30.0: AHR, 1.19 [95% CI, 1.11-1.29]; and >30.0: AHR, 1.32 [95% CI, 1.19-1.46]; *P* < .001 for trend). These findings remained unchanged after further adjusting for age and marital status at baseline, educational level, diabetes, smoking, alcohol consumption, and enrollment period (eTable 3 in Supplement 1). Additionally, the positive linear trend identified using BMI categories was consistent with the spline curves in which the risk of incident CRC was analyzed over the spectrum of BMI as a continuous variable (Figure 1A and eFigure 1A in Supplement 1). The association pattern was similar between sexes (Table 1 and Figure 1B and eFigure 1B in Supplement 1).

**Table 1.** Associations Between BMI and CRC Incidence by Sex

CRC incidence	BMI						*P* value for trend	*P* value for heterogeneity^a^
	<18.5	18.5-23.0	>23.0-25.0	>25.0-27.5	>27.5-30.0	>30.0		
**Total**								
Person-years	488 697	4 001 667	2 250 685	1 609 346	663 679	401 387	NA	NA
CRC cases, No.	564	4959	2937	2156	870	414	NA	NA
AHR (95% CI)^b^	0.91 (0.83-1.00)	0.97 (0.92-1.01)	1 [Reference]	1.09 (1.03-1.16)	1.19 (1.11-1.29)	1.32 (1.19-1.46)	<.001	NA
**Female**								
Person-years	279 117	2 157 187	1 187 946	856 971	393 975	387 810	NA	NA
CRC cases, No.	267	2030	1212	937	427	235	NA	NA
AHR (95% CI)^c^	0.96 (0.84-1.10)	1.00 (0.93-1.07)	1 [Reference]	1.09 (1.00-1.18)	1.16 (1.04-1.30)	1.24 (1.08-1.43)	<.001	NA
**Male**								
Person-years	209 581	1 844 481	1 602 739	752 375	269 705	113 577	NA	NA
CRC cases, No.	297	2929	1725	1219	443	179	NA	.05
AHR (95% CI)^c^	0.88 (0.78-1.00)	0.95 (0.89-1.01)	1 [Reference]	1.10 (1.02-1.18)	1.24 (1.11-1.37)	1.47 (1.26-1.72)	<.001	NA

Abbreviations: AHR, adjusted hazard ratio; BMI, body mass index (calculated as weight in kilograms divided by height in meters squared); CRC, colorectal cancer; NA, not applicable.

^a^ *P* value was obtained from the interaction test between BMI and sex after model adjustment.

^b^ Model adjusted for age at baseline, sex, diabetes at baseline, current smoker, and ever alcohol use.

^c^ Model adjusted for age at baseline, diabetes at baseline, current smoker, and ever alcohol use.

### BMI and Risk of CRC Mortality

**Figure 2.**
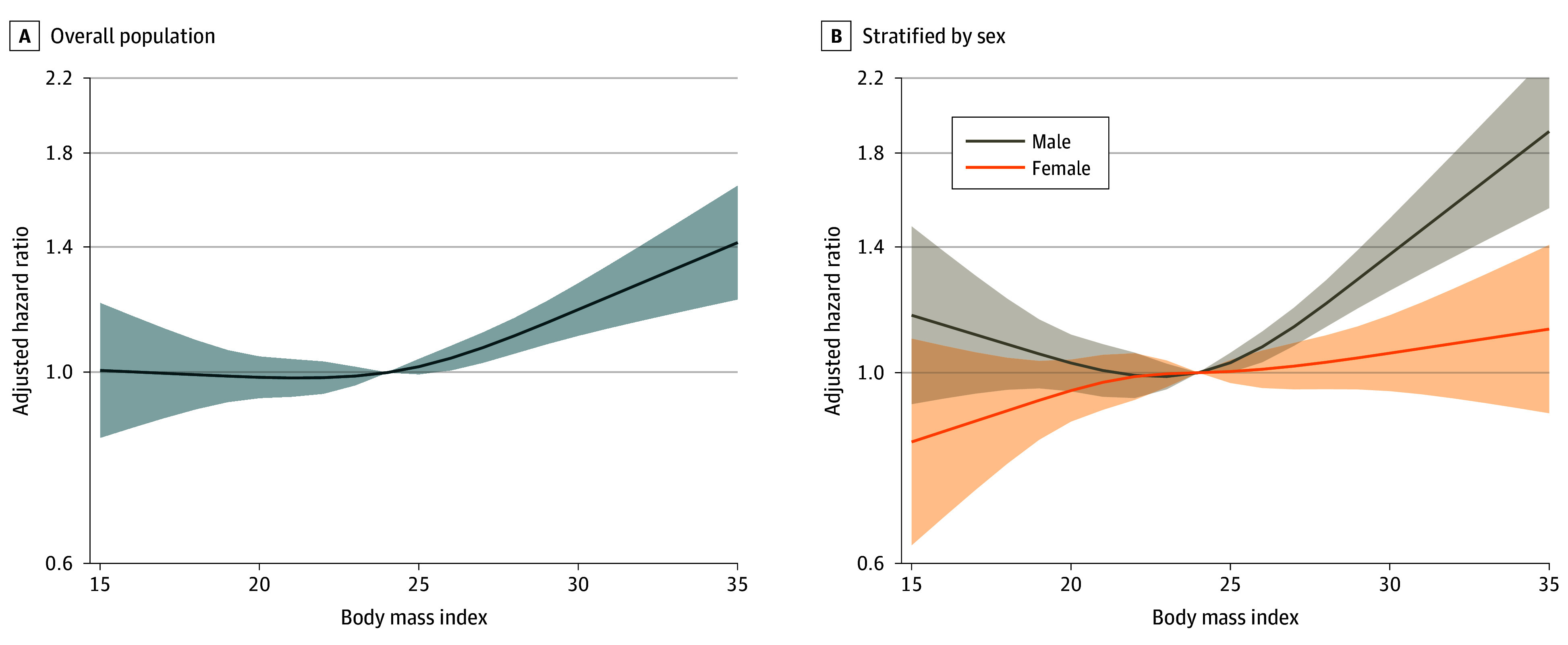
Association Between Body Mass Index and Risk of Colorectal Cancer Mortality A, Model adjusted for age at baseline, sex, diabetes at baseline, current smoker, and ever alcohol use. B, Model adjusted for age at baseline, diabetes at baseline, current smoker, and ever alcohol use. Body mass index was calculated as weight in kilograms divided by height in meters squared. Shaded areas indicate 95% CIs.

We found positive associations between individuals with a BMI greater than 27.5 and risk of CRC-related mortality compared with participants with what is considered a normal BMI (ie, >23.0-25.0). Among individuals with a BMI of greater than 27.5, the risk of CRC-related death showed a consistent increase across BMI categories ranging from 18% to 38% (>27.5-30.0: AHR, 1.18 [95% CI, 1.04-1.34] and >30.0: AHR, 1.38 [95% CI, 1.18-1.62]; *P* < .001 for trend) (Table 2 and Figure 2A). A heterogeneity between men and women for the association between BMI and CRC-related deaths is suggested (*P* = .02 for heterogeneity) (Table 2), which was also supported by sex-specific spline curves (Figure 2B and eFigure 2B in Supplement 1), and an association between BMI and CRC-related death was only found among men with a BMI of more than 30.0 (AHR, 1.87 [95% CI, 1.49-2.34]; *P* < .001 for trend) but not among women (*P* = .15 for trend). The association followed a J-shaped pattern for men, while a flat spline curve was observed for women over the spectrum of BMI as a continuous variable in both main analyses and sensitivity analyses. These findings remained unchanged after further adjusting for age and marital status at baseline, educational level, diabetes, smoking, alcohol consumption, and enrollment period (eTable 4 in Supplement 1).

**Table 2.** Associations Between BMI and CRC Mortality by Sex

CRC mortality	BMI						*P* value for trend	*P* value for heterogeneity^a^
	<18.5	18.5-23.0	>23.0-25.0	>25.0-27.5	>27.5-30.0	>30.0		
**Total**								
Person-years	511 436	4 267 499	2 411 707	1 741 818	720 981	427 652	NA	NA
CRC deaths, No.	282	1973	1071	709	332	183	NA	NA
AHR (95% CI)^b^	0.96 (0.84-1.10)	0.99 (0.92-1.07)	1 [Reference]	0.98 (0.89-1.08)	1.18 (1.04-1.34)	1.38 (1.18-1.62)	<.001	NA
**Female**								
Person-years	291 872	2 290 603	1 263 252	919 250	424 514	304 144	NA	NA
CRC deaths, No.	122	775	457	313	176	96	NA	NA
AHR (95% CI)^c^	0.93 (0.76-1.14)	1.00 (0.89-1.12)	1 [Reference]	0.95 (0.82-1.10)	1.18 (0.99-1.40)	1.10 (0.88-1.38)	.15	NA
**Male**								
Person-years	219 565	1 976 897	1 148 456	822 568	296 468	123 508	NA	NA
CRC deaths, No.	160	1198	614	396	156	87	NA	.02
AHR (95% CI)^c^	0.99 (0.83-1.18)	0.98 (0.89-1.09)	1 [Reference]	1.00 (0.88-1.14)	1.18 (0.99-1.41)	1.87 (1.49-2.34)	<.001	NA

Abbreviations: AHR, adjusted hazard ratio; BMI, body mass index (calculated as weight in kilograms divided by height in meters squared); CRC, colorectal cancer; NA, not applicable.

^a^ *P* value was obtained from the interaction test between BMI and sex after model adjustment.

^b^ Model adjusted for age at baseline, sex, diabetes at baseline, current smoker, and ever alcohol use.

^c^ Model adjusted for age at baseline, diabetes at baseline, current smoker, and ever alcohol use.

### Incidence and Mortality of CRC Subsites

For incidence and mortality analyses according to subsites, the findings were in line with what was described above when colon cancer, rectal cancer, and unknown CRC groups were combined as CRC. Risk of CRC incidence and mortality associated with BMI was greater among individuals with colon cancer (eTables 5-8 in Supplement 1).

### Stratified Analyses by Geographic Location

Regarding the association between BMI and CRC incidence, our analysis showed no difference across geographic locations (Table 3). No association was found between BMI categories and geographic subgroups, suggesting that the association between BMI and CRC incidence and mortality was homogeneous across subpopulations.

**Table 3.** Associations Between BMI and CRC Incidence in Subgroups

Subgroup	BMI						*P* value for trend	*P* value for heterogeneity^a^
	<18.5	18.5-23.0	>23.0-25.0	>25.0-27.5	>27.5-30.0	>30.0		
**Geographic location**								
China (n = 217 190)								
CRC cases, No.	190	1476	940	697	330	141	NA	NA
MVHR (95% CI)^b^	0.95 (0.81-1.12)	1.02 (0.94-1.11)	1 [Reference]	1.11 (1.00-1.22)	1.26 (1.11-1.42)	1.15 (0.96-1.37)	<.001	NA
South Korea (n = 58 201)								
CRC cases, No.	7	106	77	79	28	11	NA	NA
MVHR (95% CI)^b^	0.61 (0.28-1.33)	0.89 (0.66-1.20)	1 [Reference]	1.24 (0.90-1.69)	1.14 (0.74-1.76)	1.28 (0.68-2.42)	.01	.32
Japan (n = 294 592)								
CRC cases, No.	364	3356	1906	1357	491	217	NA	NA
MVHR (95% CI)^b^	0.92 (0.82-1.03)	0.95 (0.90-1.01)	1 [Reference]	1.08 (1.00-1.15)	1.15 (1.04-1.27)	1.37 (1.19-1.58)	<.001	NA
Iran (n = 49 998)								
CRC cases, No.	3	21	14	23	21	45	NA	NA
MVHR (95% CI)^b^	0.65 (0.19-2.28)	0.90 (0.46-1.78)	1 [Reference]	1.28 (0.66-2.48)	1.41 (0.72-2.79)	2.19 (1.19-4.04)	<.001	NA
**Diabetes **								
Without (n = 574 755)								
CRC cases, No.	524	4568	2683	1947	782	361	NA	NA
MVHR (95% CI)^c^	0.93 (0.84-1.02)	0.97 (0.92-1.01)	1 [Reference]	1.08 (1.02-1.15)	1.20 (1.11-1.30)	1.31 (1.17-1.47)	<.001	.40
With (n = 35 153)								
CRC cases, No.	16	282	221	181	82	47	NA	NA
MVHR (95% CI)^c^	0.59 (0.35-0.98)	0.96 (0.80-1.14)	1 [Reference]	1.14 (0.94-1.39)	1.13 (0.88-1.46)	1.31 (0.95-1.82)	.002	NA
**Smoking status**								
Former or never smoker (n = 440 424)								
CRC cases, No.	318	3022	1970	1518	662	331	NA	NA
MVHR (95% CI)^d^	0.91 (0.81-1.03)	0.98 (0.93-1.04)	1 [Reference]	1.09 (1.02-1.16)	1.23 (1.13-1.35)	1.34 (1.19-1.51)	<.001	.58
Current smoker (n = 174 204)								
CRC cases, No.	235	1889	950	626	204	82	NA	NA
MVHR (95% CI)^d^	0.92 (0.79-1.06)	0.94 (0.87-1.01)	1 [Reference]	1.11 (1.00-1.23)	1.10 (0.94-1.28)	1.29 (1.03-1.62)	<.001	NA
**Alcohol use status**								
Never alcohol user (n = 394 664)								
CRC cases, No.	328	2823	1682	1328	580	299	NA	NA
MVHR (95% CI)^e^	0.98 (0.87-1.11)	1.01 (0.95-1.07)	1 [Reference]	1.12 (1.04-1.20)	1.23 (1.12-1.35)	1.34 (1.18-1.51)	<.001	.27
Ever alcohol user (n = 194 493)								
CRC cases, No.	202	1819	1127	740	272	106	NA	NA
MVHR (95% CI)^e^	0.88 (0.76-1.03)	0.90 (0.84-0.98)	1 [Reference]	1.03 (0.94-1.13)	1.16 (1.01-1.32)	1.27 (1.04-1.56)	<.001	NA

Abbreviations: BMI, body mass index (calculated as weight in kilograms divided by height in meters squared); CRC, colorectal cancer; MVHR, multivariable hazard ratio; NA, not applicable.

^a^ *P* value was obtained from the interaction test between BMI and subgroup after model adjustment.

^b^ Model adjusted for age at baseline, sex, diabetes at baseline, current smoker, and ever alcohol use.

^c^ Model adjusted for age at baseline, sex, current smoker, and ever alcohol use.

^d^ Model adjusted for age at baseline, sex, diabetes at baseline, and ever alcohol use.

^e^ Model adjusted for age at baseline, sex, diabetes at baseline, and current smoker.

We found a positive association between BMI and CRC-related mortality in China, Japan, and Iran. On the other hand, this significant trend was not present in South Korea. However, the patterns of association between BMI and CRC-related mortality did not show significant difference across geographic locations (eTable 9 in Supplement 1).

### Stratified Analysis by Diabetes, Smoking, and Alcohol Use

Overall, the positive associations between BMI and risk of incident CRC and risk of death from CRC were mostly found in stratified analyses by diabetes, current smoking status, and ever alcohol use (eTables 10 and 11 in Supplement 1). Given the potential heterogeneity between men and women, we also explored a possible sex difference for the examined associations in various subgroups (eTables 12-15 in Supplement 1). Specifically, in the analysis of participants with complete data on covariates available, the risk of CRC-related mortality appeared to be more pronounced for men than for women among current smokers and those who consume alcohol. It should be noted that smoking was far less common in women than in men (current smokers constituted 5.8% of female and 51.0% of male participants).

We further conducted a sensitivity analysis by comparing results between cohorts with an actual measurement of BMI and cohorts with self-reported BMI. There was no distinguishable difference in the association between BMI and CRC incidence and mortality using the 2 methods of BMI measurements (eTables 16-19 in Supplement 1).

## Discussion

This cohort study of 619 981 Asian participants assessed for CRC incidence and 650 195 Asian participants assessed for CRC-related mortality showed a positive association between BMI and risks of CRC incidence and CRC-related mortality. Risk of CRC-related mortality associated with BMI was greater among men with a BMI of more than 30.0 than among women, and risk of CRC incidence and mortality associated with BMI was more prominent among individuals with colon cancer.

Obesity has been associated with increased risk and mortality of many types of cancers, including CRC.^30,31^ The mutagenic capability of obesity using peroxidation pathways as well as a dysregulated metabolism may promote tumor development.^32^ Specifically, an altered metabolism of glucose and increased insulin secretion may be responsible for the increased risk of CRC in people with obesity.^33^ This effect is mainly modulated by insulin as a mitogen and tumor growth promoter.^34^ In addition, the low-grade chronic inflammatory status stimulates secretion of signaling cytokines that are involved in initiation, progression, and metastasis of CRC.^31^ Moreover, as a state of nutrient excess, obesity promotes neoplastic transformation using activating cellular growth pathways.^35^ In the current study, we detected a positive association between BMI and CRC incidence among individuals who were both diabetic and nondiabetic. This finding may partly explain the association of BMI with CRC development, independent of an altered glucose metabolic pathway. Compared with North Americans and Europeans, East Asians are leaner and tend to develop diabetes and glucose intolerance at lower BMI levels.^36,37^ Therefore, to better understand the impact of obesity and the potential impact of the altered glucose metabolism among Asian individuals, BMI cutoff points that are consistent with those of the ACC should be applied.

One main finding in our study was an association between BMI and risk of CRC-related death among men with a BMI of more than 30.0. The extrapolation of BMI on survival of CRC cases and the disparity between men and women are subjects of ongoing debate. Similar to our findings, a systematic review reported that prediagnostic obesity (but not overweight) was associated with increased risk of mortality among men only.^38^ The differential distribution of adipose tissue may partly explain the higher risk to tumorigenesis and cancer mortality in men. Men have a predilection to develop central adiposity rather than generalized obesity, which is more common in women.^39^ Central adiposity has been reported to be strongly correlated with hyperinsulinoma and more intense insulin resistance.^40^ This association is prominent among Asians, as central obesity is an established risk factor for type 2 diabetes in addition to BMI.^41^ Furthermore, excessive visceral adipose tissue distribution is an established risk factor for comorbid states such as type 2 diabetes, which increases the risk of colon cancer. The comparative studies have been between European or American and Asian populations and reported a distinguished pattern in adipose tissue^42^ and risk of insulin resistance in relation to a visceral tissue compartment.^43^ It is noteworthy that the sex differential association between higher BMI and CRC mortality may be partially explained by incomplete adjustment or residual confounding by smoking and alcohol consumption among women. Therefore, the differential confounder adjustment may be partly responsible for the finding regarding CRC mortality among men and women.

Another explanation for our primary finding is the potential association between hormone replacement therapy (HRT) and BMI and the risk of CRC-related death in women. A meta-analysis of 5 cohorts, including 10 013 survivors of CRC, found an inverse association between current use of HRT and CRC-specific mortality and overall mortality.^44^ Additionally, Asian women have a relatively low prevalence of HRT use,^45^ which must be considered in relation to global trends. The other reason might be a higher adherence of women to screening.^46^ While the current study adjusted for smoking and alcohol use status, detailed quantifiable data on smoking or alcohol consumption may be crucial in assessing the risk of mortality. Men also smoke and consume more alcohol than women.^47^

The association between BMI and CRC-specific mortality has been reported in different studies worldwide.^16,31,48^ Moreover, insulin resistance is an established factor associated with decreased survival in CRC.^49^ In the current study, the positive association between BMI and risk of CRC-related mortality was present in subpopulations of China, Iran, and Japan. The absence of a significant trend in CRC-related mortality, specifically in South Korea, may be partly explained by the limited number of recorded deaths in the South Korean participating cohort. The other countries that had significantly positive trends were China and Iran, in which the Golestan cohort study, for instance, was associated with either being overweight or obese (BMI ≥27.5). However, the overall patterns of association between BMI and CRC-related mortality were comparable across geographic locations.

A study by Croft et al^50^ reported that among 4 components of metabolic syndrome (ie, overweight or obesity, diabetes, hypertension, and dyslipidemia), only diabetes was associated with progression-free survival, while obesity, dyslipidemia, and hypertension did not show a similar association with the outcome. The lack of a significant difference in mortality among the diabetic and nondiabetic strata may be attributable to the lack of power.

### Strengths and Limitations

Our study has several strengths. This study benefited from the prospective design that limited recall bias. Body mass index was stratified using ACC-based cutoffs, which made the clinical interpretations more relevant for Asian populations. Furthermore, the analysis of individual-level data from large multicenter cohorts allowed us to evaluate the associations between BMI and risk of CRC incidence and mortality with standardized categorization of exposure and confounding variables. We could separately investigate tumors located in the colon or the rectum. Furthermore, the large sample size allowed us to conduct sensitivity analyses to evaluate the reverse causality, thus strengthening the rigorousness of our study.

Our study also has several limitations. First, the anthropometric data acquisition in some cohorts was based on self-reported forms, although the validation of the self-reported BMI, height, and weight was high among these cohorts.^12,51^ Second, there was a lack of longitudinal measurement of BMI, as our analyses were based on a baseline measurement of BMI. Therefore, the current study could not evaluate the impact of subsequent changes in BMI. Third, our analyses might have effects of residual confounding, as we did not control for other important confounding factors in the multivariable models, such as physical activity, socioeconomic status, or family history of CRC. Furthermore, the tumor stage and treatment regimen were not captured at the time of enrollment. While overall the study enrollment was focused on the early stage of the disease, these factors may play a partial role in the outcome of participants.

## Conclusions

This cohort study using data derived from the ACC demonstrated positive associations between BMI and risk of incident CRC and related deaths. These risks were more apparent in participants with colon cancer and were greater in men. Findings from this study may have substantial implications regarding our understanding of the burden of BMI on CRC incidence and deaths in Asian populations.

## Notes

Supplement 1.
eMethods
**eFigure 1.** Associations Between BMI and CRC Incidence **eFigure 2.** Associations Between BMI and CRC Mortality **eTable 1.** Characteristics of Participating Cohorts in The Asia Cohort Consortium for CRC Incidence Analysis **eTable 2.** Characteristics of Participating Cohorts in The Asia Cohort Consortium for CRC Mortality Analysis **eTable 3.** Associations Between BMI and CRC Incidence During Follow-up by Sex Among Those Having Complete Data on Covariates **eTable 4.** Associations Between BMI and CRC Mortality During Follow-up by Sex Among Those Having Complete Data on Covariates **eTable 5.** Associations Between BMI and CRC Histologic Subtypes Incidence During Follow-up by Sex **eTable 6.** Associations Between BMI and CRC Subtypes Incidence by Sex Among Those With Complete Data on Covariates **eTable 7.** Associations Between BMI and CRC Histologic Subtypes Mortality by Sex **eTable 8.** Associations Between BMI and CRC Histologic Subtypes Mortality by Sex Among Those With Complete Data on Covariates **eTable 9.** Associations Between BMI and CRC Mortality in Subgroups Among Those Having Complete Data on Covariates **eTable 10**. Associations Between BMI and CRC Incidence in Subgroups Among Those With Complete Data on Covariates **eTable 11**. Associations Between BMI and CRC Mortality in Subgroups **eTable 12**. Associations Between BMI and CRC Incidence by Sex in Subgroups **eTable 13**. Associations Between BMI and CRC Incidence by Sex in Subgroups Among Those With Complete Data on Covariates **eTable 14**. Associations Between BMI and CRC Mortality by Sex in Subgroups **eTable 15**. Associations Between BMI and CRC Mortality by Sex in Subgroups Among Those With Complete Data on Covariates **eTable 16.** Associations Between BMI (Actual Measurement) and CRC Incidence by Sex **eTable 17.** Associations Between BMI (Self-Report) and CRC Incidence by Sex **eTable 18.** Associations Between BMI (Actual Measurement) and CRC Mortality by Sex **eTable 19.** Associations Between BMI (Self-Report) and CRC Mortality by Sex

Supplement 2.
Data Sharing Statement
