# Supplementary material for: Body Mass Index and Risk of Colorectal Cancer Incidence and Mortality in Asia
Source: JAMA Netw Open. 2024 Aug 28;7(8):e2429494. doi: 10.1001/jamanetworkopen.2024.29494 (PMC11358861; doi:10.1001/jamanetworkopen.2024.29494)
Supplement: Supplement 1. — eMethods eFigure 1. Associations Between BMI and CRC Incidence eFigure 2. Associations Between BMI and CRC Mortality eTable 1. Characteristics of Participating Cohorts in The Asia Cohort Consortium for CRC Incidence Analysis eTable 2. Characteristics of Participating Cohorts in The Asia Cohort Consortium for CRC Mortality Analysis eTable 3. Associations Between BMI and CRC Incidence During Follow-up by Sex Among Those Having Complete Data on Covariates eTable 4. Associations Between BMI and CRC Mortality During Follow-up by Sex Among Those Having Complete Data on Covariates eTable 5. Associations Between BMI and CRC Histologic Subtypes Incidence During Follow-up by Sex eTable 6. Associations Between BMI and CRC Subtypes Incidence by Sex Among Those With Complete Data on Covariates eTable 7. Associations Between BMI and CRC Histologic Subtypes Mortality by Sex eTable 8. Associations Between BMI and CRC Histologic Subtypes Mortality by Sex Among Those With Complete Data on Covariates eTable 9. Associations Between BMI and CRC Mortality in Subgroups Among Those Having Complete Data on Covariates eTable 10. Associations Between BMI and CRC Incidence in Subgroups Among Those With Complete Data on Covariates eTable 11. Associations Between BMI and CRC Mortality in Subgroups eTable 12. Associations Between BMI and CRC Incidence by Sex in Subgroups eTable 13. Associations Between BMI and CRC Incidence by Sex in Subgroups Among Those With Complete Data on Covariates eTable 14. Associations Between BMI and CRC Mortality by Sex in Subgroups eTable 15. Associations Between BMI and CRC Mortality by Sex in Subgroups Among Those With Complete Data on Covariates eTable 16. Associations Between BMI (Actual Measurement) and CRC Incidence by Sex eTable 17. Associations Between BMI (Self-Report) and CRC Incidence by Sex eTable 18. Associations Between BMI (Actual Measurement) and CRC Mortality by Sex eTable 19. Associations Between BMI (Self-Report) and CRC Mortality by Sex [file jamanetwopen-e2429494-s001.pdf]

Supplemental Online Content

Paragomi P, Zhang Z, Abe SK, et al. Body mass index and risk of colorectal cancer incidence and mortality in Asia. *JAMA Netw Open*. 2024;7(8):e2429494. doi:10.1001/jamanetworkopen.2024.29494

eMethods
eFigure 1. Associations Between BMI and CRC Incidence
eFigure 2. Associations Between BMI and CRC Mortality
eTable 1. Characteristics of Participating Cohorts in The Asia Cohort Consortium for CRC Incidence Analysis
eTable 2. Characteristics of Participating Cohorts in The Asia Cohort Consortium for CRC Mortality Analysis
eTable 3. Associations Between BMI and CRC Incidence During Follow-up by Sex Among Those Having Complete Data on Covariates
eTable 4. Associations Between BMI and CRC Mortality During Follow-up by Sex Among Those Having Complete Data on Covariates
eTable 5. Associations Between BMI and CRC Histologic Subtypes Incidence During Follow-up by Sex
eTable 6. Associations Between BMI and CRC Subtypes Incidence by Sex Among Those With Complete Data on Covariates
eTable 7. Associations Between BMI and CRC Histologic Subtypes Mortality by Sex
eTable 8. Associations Between BMI and CRC Histologic Subtypes Mortality by Sex Among Those With Complete Data on Covariates
eTable 9. Associations Between BMI and CRC Mortality in Subgroups Among Those Having Complete Data on Covariates
eTable 10. Associations Between BMI and CRC Incidence in Subgroups Among Those With Complete Data on Covariates
eTable 11. Associations Between BMI and CRC Mortality in Subgroups
eTable 12. Associations Between BMI and CRC Incidence by Sex in Subgroups
eTable 13. Associations Between BMI and CRC Incidence by Sex in Subgroups Among Those With Complete Data on Covariates
eTable 14. Associations Between BMI and CRC Mortality by Sex in Subgroups
eTable 15. Associations Between BMI and CRC Mortality by Sex in Subgroups Among Those With Complete Data on Covariates
eTable 16. Associations Between BMI (Actual Measurement) and CRC Incidence by Sex
eTable 17. Associations Between BMI (Self-Report) and CRC Incidence by Sex
eTable 18. Associations Between BMI (Actual Measurement) and CRC Mortality by Sex
eTable 19. Associations Between BMI (Self-Report) and CRC Mortality by Sex

This supplemental material has been provided by the authors to give readers additional information about their work.

© 2024 Paragomi P et al. *JAMA Network Open*.

eMethods

Study coordination

The ACC Coordinating Center provided harmonized data for this analysis, along with the data dictionary. The pooled analysis for the current study was approved by the ACC Executive Committee and by the ethical committee of the National Cancer Center Japan (No. 2014-041).

Data Harmonization

The principal investigator (PI) for each ACC collaborative project submitted study proposal to the ACC Coordinating Center where it was reviewed by the ACC Executive Committee. Upon the approval of the study proposal by the ACC Executive Committee, the ACC Coordinating Center started to consolidate data for the proposed project by inviting all ACC cohorts to participate into the approved project. The participating cohorts would then submit relevant data, including the most recent follow-up information, if possible. Once receiving data from all participating cohorts for the proposed project, the ACC Coordinating Center conducted data updating, cleaning and pooling so that only relevant data for the proposed project was ready to be further analyzed. The Principal Investigator of that approved project would also submit the Data Use Agreement Form and the Remote Access Application Form to the ACC Coordinating Center. The ACC Coordinating Center would open a remote access account for each Principal Investigator based on information provided by the PI, including IP addresses and a proxy server. Subsequently, the PI can access the ACC Data Center via Virtual Private Network (VPN) to conduct data analysis using datasets provided by the ACC Coordinating Center.

© 2024 Paragomi P et al. *JAMA Network Open*.

eFigure 1. Associations Between BMI and CRC Incidence

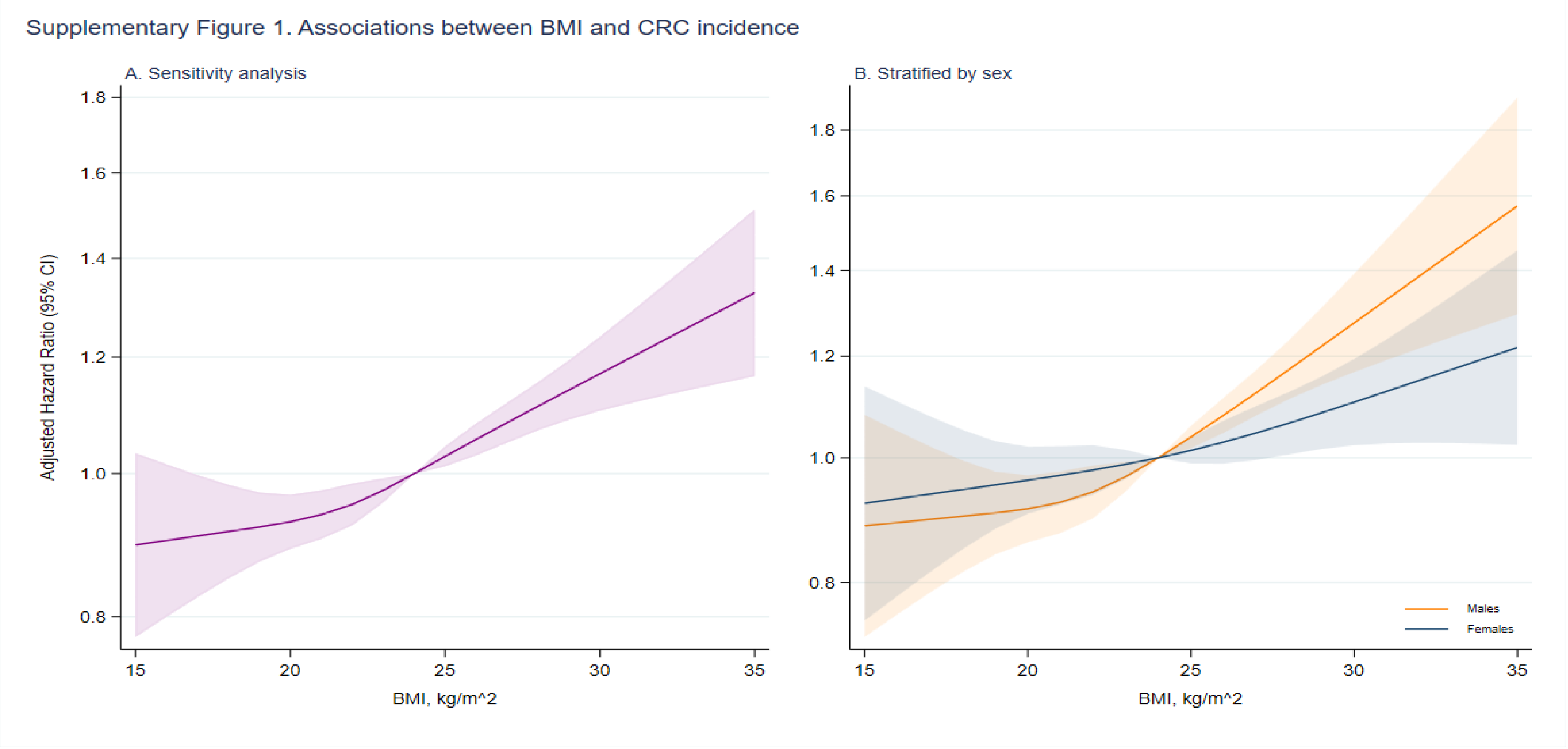

eFigure 1- Spline curves depicting the association between BMI and risk of incident colorectal cancer (CRC) in case of complete dataset of covariates.
A Model adjusted for age at baseline, sex, diabetes at baseline, current smoking, and ever alcohol use
B. Model adjusted for age at baseline, diabetes at baseline, current smoking, and ever alcohol use

© 2024 Paragomi P et al. *JAMA Network Open*.

eFigure 2. Associations Between BMI and CRC Mortality

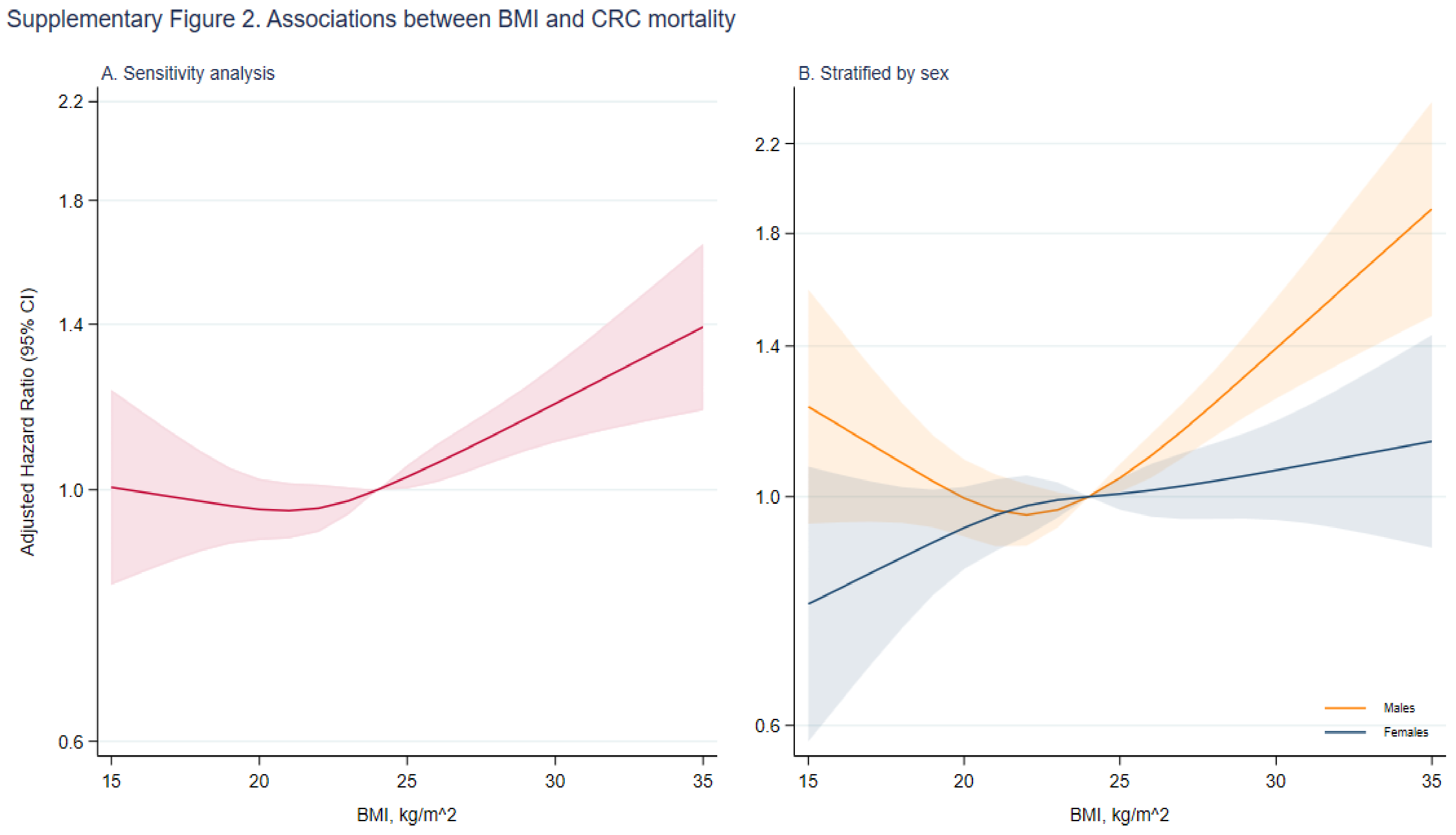

eFigure 2- Spline curves depicting the association between BMI and risk of CRC related mortality in case of complete dataset of covariates.
A Model adjusted for age at baseline, sex, diabetes at baseline, current smoking, and ever alcohol use
B. Model adjusted for age at baseline, diabetes at baseline, current smoking, and ever alcohol use

© 2024 Paragomi P et al. *JAMA Network Open*.

eTable 1. Characteristics of Participating Cohorts in The Asia Cohort Consortium for CRC Incidence Analysis

Participating cohorts	Person-year	Number of participants	Enrollment period	Follow-up duration (Median (IQR))	Age at enrollment (Mean±SD)	Male (%)	CRC cases	BMI (kg/m^2, %)					
								< 18.5	18.5 - 23	23 - 25	25 - 27.5	27.5 - 30	≥ 30
**Mainland China**													
SCS	400 765	18 076	1986-1989	25.5 (16.5-28.7)	55.8±5.7	100.0	648	9.6	53.0	20.2	12.6	3.5	1.0
SMHS	731 343	61 267	2001-2006	12.2 (11.2-13.5)	55.3±9.7	100.0	673	4.2	36.6	26.0	22.6	8.0	2.6
SWHS	1 300 475	74 802	1996-2000	18.1 (17.2-18.8)	52.6±9.1	0.0	1,077	3.4	37.5	23.6	20.6	9.7	5.2
**Japan**													
JPHC1	891 912	42 411	1990-1992	22.6 (22.3-22.8)	49.6±5.9	47.8	1,588	2.6%	42.2%	26.7%	19.0%	6.8%	2.7%
JPHC2	976 595	55 007	1992-1995	19.7 (18.9-19.8)	54.2±8.8	47.4	1,632	3.6%	43.3%	25.5%	18.1%	6.7%	2.8%
Miyagi	801 072	37 367	1990	24.6 (21.0-24.6)	51.4±7.6	55.0	1,308	2.5%	42.2%	26.9%	18.7%	7.1%	2.5%
Ohsaki	426 751	39 484	1995	13.2 (9.9-13.2)	59.5±10.4	53.7	685	4.0%	42.3%	25.8%	18.2%	6.9%	2.7%
LSS	1 090 376	49 781	1963-1993	21.9 (10.9-32.0)	52.2±13.7	39.7	1,488	13.3%	53.9%	17.2%	10.3%	3.5%	1.9%
Takayama	404 496	29 569	1992	15.6 (15.6-15.6)	55.4±12.7	45.7	591	8.6%	54.6%	21.7%	11.1%	3.1%	0.9%
3 Pref. Miyagi	232 516	20 435	1984	15.0 (7.4-15.0)	56.3±11.3	47.9	156	5.2%	45.7%	24.4%	16.5%	5.8%	2.4%
3 Pref. Aichi	238 523	20 538	1985	15.2 (7.6-15.2)	54.4±10.8	50.4	243	8.9%	55.8%	21.3%	10.4%	2.8%	0.9%
**South Korea**													
KMCC	263 390	18 933	1993-2004	13.7 (10.9-17.4)	53.7±14.4	40.0	222	4.5%	40.9%	23.5%	19.7%	8.0%	3.4%
KNCC	366 667	39 268	2002-2015	9.3 (6.6-12.1)	49.9±9.3	50.7	86	2.4%	37.6%	26.9%	22.3%	7.9%	2.9%
**Singapore**													
SCHS	876 339	63 045	1993-1999	15.2 (12.6-16.7)	56.5±8.0	44.2	1,376	6.4%	41.5%	30.2%	13.2%	5.6%	3.1%
**Iran**													
Golestan	622 555	49 998	2004-2008	13.0 (12.0-14.1)	52.1±8.9	42.4	127	4.8%	22.1%	13.8%	18.2%	15.8%	25.4%
**Total**	**9 623 775**	**619 981**	**1963-2015**	**15.2 (12.1-19.2)**	**53.8±10.1**	**48.0**	**11,900**	**5.3%**	**41.6%**	**23.9%**	**17.3%**	**7.3%**	**4.6%**

CRC: colorectal cancer, IQR: interquartile range, SD: standard deviation, BMI: body mass index, SCS: Shanghai Cohort Study, SMHS: Shanghai Men's Health Study, SWHS: Shanghai Women's Health Study, JPHC: Japan Public Health Center-based Prospective Study, Miyagi: Miyagi Cohort Study, Ohsaki: Ohsaki National Health Insurance Cohort Study, LSS: Life Span Study - Radiation Effects Research Foundation, Takayama: Takayama Study, 3 Pref. Miyagi: Three Prefecture Cohort Study Miyagi, 3 Pref. Aichi: Three Prefecture Cohort Study Aichi, KMCC: Korean Multi-center Cancer Cohort Study, KNCC: Korean National Cancer Center Cohort, SCHS: Singapore Chinese Health Study, Golestan: Golestan Cohort Study

© 2024 Paragomi P et al. *JAMA Network Open*.

eTable 2. Characteristics of Participating Cohorts in The Asia Cohort Consortium for CRC Mortality Analysis

Participating cohorts	Person-year	Number of participants	Enrollment period	Follow-up duration (Median (IQR))	Age at enrollment (Mean±SD)	Male (%)	CRC deaths	BMI (kg/m^2)					
								< 18.5	18.5 - 23	23 - 25	25 - 27.5	27.5 - 30	≥ 30
**Mainland China**													
SCS	40 ,220	18 099	1986-1989	25.5 (16.5-28.7)	55.8±5.7	100.0%	417	9.6%	53.0%	20.2%	12.6%	3.5%	1.0%
SMHS	732 585	61 397	2001-2006	12.2 (11.2-13.5)	55.4±9.7	100.0%	348	4.2%	36.6%	26.0%	22.6%	8.0%	2.6%
SWHS	1 301 395	74 859	1996-2000	18.1 (17.2-18.8)	52.6±9.1	0.0%	508	3.4%	37.5%	23.6%	20.6%	9.7%	5.2%
**Japan**													
JPHC1	893 892	42 528	1990-1992	22.6 (22.3-22.8)	49.6±5.9	47.8%	337	2.6%	42.2%	26.7%	19.0%	6.8%	2.7%
JPHC2	979 639	55 218	1992-1995	19.7 (18.8-19.8)	54.2±8.8	47.5%	451	3.6%	43.3%	25.6%	18.1%	6.7%	2.8%
Miyagi	806 479	37 663	1990	24.6 (20.9-24.6)	51.4±7.6	55.1%	266	2.5%	42.2%	26.9%	18.8%	7.1%	2.5%
Ohsaki	429 922	39 757	1995	13.2 (9.9-13.2)	59.5±10.4	53.9%	221	4.0%	42.3%	25.8%	18.2%	6.9%	2.7%
LSS	1 090 342	49 754	1963-1993	21.9 (10.9-32.0)	52.2±13.7	39.7%	700	13.3%	53.8%	17.2%	10.3%	3.5%	1.9%
Takayama	405 475	29 661	1992	15.6 (15.6-15.6)	55.4±12.7	45.8%	202	8.6%	54.6%	21.7%	11.1%	3.1%	0.9%
3 Pref. Miyagi	232 957	20 470	1984	15.0 (7.4-15.0)	56.3±11.3	47.9%	115	5.2%	45.7%	24.4%	16.5%	5.8%	2.4%
3 Pref. Aichi	238 509	20 529	1985	15.2 (7.7-15.2)	54.4±10.8	50.5%	107	8.9%	55.8%	21.3%	10.4%	2.8%	0.9%
**South Korea**													
KMCC	264 162	19 006	1993-2004	13.7 (10.9-17.4)	53.7±14.4	40.1%	81	4.5%	41.0%	23.5%	19.7%	8.0%	3.4%
KNCC	367 590	39 369	2002-2015	9.3 (6.6-12.1)	49.9±9.3	50.7%	4	2.4%	37.6%	26.9%	22.3%	7.9%	2.9%
**Singapore**													
SCHS	877 778	63 145	1993-1999	15.2 (12.6-16.7)	56.5±8.0	44.2%	617	6.4%	41.6%	30.2%	13.2%	5.6%	3.1%
**Iran**													
Golestan	622 742	50 018	2004-2008	13.0 (12.0-14.1)	52.1±8.9	42.4%	80	4.8%	22.1%	13.8%	18.2%	15.8%	25.4%
**Taiwan**													
CBCSP	359 851	23 601	1991-1992	15.9 (15.5-16.3)	47.3±10.0	50.3%	71	3.2%	37.2%	23.7%	21.3%	9.8%	4.8%
CVDFACTS	76 551	5 121	1990-1993	15.7 (14.9-16.7)	47.5±15.5	44.1%	25	5.0%	40.1%	22.6%	19.0%	8.8%	4.6%
**Total**	10 081 089	650 195	1963-2015	15.3 (12.2-18.9)	53.5±10.2	48.1%	4,550	5.2%	41.4%	23.9%	17.5%	7.4%	4.6%

CRC: colorectal cancer, IQR: interquartile range, SD: standard deviation, BMI: body mass index, SCS: Shanghai Cohort Study, SMHS: Shanghai Men's Health Study, SWHS: Shanghai Women's Health Study, JPHC: Japan Public Health Center-based Prospective Study, Miyagi: Miyagi Cohort Study, Ohsaki: Ohsaki National Health Insurance Cohort Study, LSS: Life Span Study - Radiation Effects Research Foundation, Takayama: Takayama Study, 3 Pref. Miyagi: Three Prefecture Cohort Study Miyagi, 3 Pref. Aichi: Three Prefecture Cohort Study Aichi, KMCC: Korean Multi-center Cancer Cohort Study, KNCC: Korean National Cancer Center Cohort, SCHS: Singapore Chinese Health Study, Golestan: Golestan Cohort Study, CBCSP: Community-based Cancer Screening Project, CVDFACTS: Cardiovascular Diseases Risk Factor Two-Township Study

© 2024 Paragomi P et al. *JAMA Network Open*.

eTable 3. Associations Between BMI and CRC Incidence During Follow-up by Sex Among Those Having Complete Data on Covariates

	BMI (kg/m^2)							
	**< 18.5**	**18.5 - 23**	**23 - 25**	**25 - 27.5**	**27.5 - 30**	**≥ 30**	***P for trend***	$P_{heterogeneity}$[c]
**Total**								
CRC cases	444	3398	1940	1406	577	291		
MVHR (95% CI)[a]	0.91 (0.82 - 1.01)	0.97 (0.92 - 1.03)	Ref.	1.09 (1.02 - 1.17)	1.18 (1.08 - 1.30)	1.31 (1.15 - 1.48)	<.001	
**Males**								
CRC cases	234	1982	1093	770	273	116		0.179
MVHR (95% CI)[b]	0.88 (0.76 - 1.01)	0.95 (0.88 - 1.02)	Ref.	1.11 (1.01 - 1.22)	1.20 (1.05 - 1.37)	1.45 (1.19 - 1.76)	<.001	
**Females**								
CRC cases	210	1416	847	636	304	175		
MVHR (95% CI)[b]	0.97 (0.83 - 1.13)	1.02 (0.93 - 1.11)	Ref.	1.07 (0.97 - 1.19)	1.18 (1.03 - 1.34)	1.26 (1.05 - 1.47)	0.002	

BMI: body mass index, CRC: colorectal cancer

[a]Model adjusted for age at baseline, sex, education, marital status, diabetes at baseline, current smoking, ever alcohol use, and enrollment period

[b]Model adjusted for age at baseline, education, marital status, diabetes at baseline, current smoking, ever alcohol use, and enrollment period

[c]P value was obtained form the interaction test between BMI and sex after model adjustment

© 2024 Paragomi P et al. *JAMA Network Open*.

eTable 4. Associations Between BMI and CRC Mortality During Follow-up by Sex Among Those Having Complete Data on Covariates

	BMI (kg/m^2)							
	**< 18.5**	**18.5 - 23**	**23 - 25**	**25 - 27.5**	**27.5 - 30**	**≥ 30**	***P for trend***	***P$_{heterogeneity}$***[c]
**Total**								
Person-years	419,181	3,156,570	1,760,317	1,288,383	558,787	363,401		
CRC deaths	235	1480	803	539	261	147		
MVHR (95% CI)[a]	0.93 (0.81 - 1.08)	0.96 (0.88 - 1.05)	Ref.	1.00 (0.89 - 1.11)	1.20 (1.04 - 1.38)	1.35 (1.13 - 1.62)	<.001	
**Males**								
Person-years	181,355	1,450,597	809,158	595,082	223,532	99,398		
CRC deaths	136	893	438	298	116	66		0.044
MVHR (95% CI)[b]	0.98 (0.81 - 1.20)	0.96 (0.86 - 1.08)	Ref.	1.05 (0.91 - 1.22)	1.19 (0.97 - 1.46)	1.86 (1.43 - 2.42)	<.001	
**Females**								
	237,827	1,705,974	951,160	693,302	335,256	264,003		
CRC deaths	99	587	365	241	145	81		
MVHR (95% CI)[b]	0.87 (0.70 - 1.10)	0.97 (0.85 - 1.11)	Ref.	0.93 (0.79 - 1.10)	1.19 (0.98 - 1.45)	1.08 (0.85 - 1.39)	0.072	

BMI: body mass index, CRC: colorectal cancer

[a]Model adjusted for age at baseline, sex, education, marital status, diabetes at baseline, current smoking, ever alcohol use, and enrollment period

[b]Model adjusted for age at baseline, education, marital status, diabetes at baseline, current smoking, ever alcohol use, and enrollment period

[c]P value was obtained form the interaction test between BMI and sex after model adjustment

© 2024 Paragomi P et al. *JAMA Network Open*.

eTable 5. Associations Between BMI and CRC Histologic Subtypes Incidence During Follow-up by Sex

	BMI (kg/m^2)							
	< 18.5	18.5 - 23	23 - 25	25 - 27.5	27.5 - 30	≥ 30	*P for trend*	$P_{heterogeneity}$[c]
**Topography: colon cancer**								
**Total**								
Person-years	495,851	4,086,813	2,298,451	1,642,336	675,794	405,873		
Colon cancer cases	349	3011	1878	1380	567	286		
MVHR (95% CI)[a]	0.91 (0.81 - 1.03)	0.94 (0.89 - 1.00)	Ref.	1.08 (1.01 - 1.16)	1.21 (1.10 - 1.33)	1.38 (1.22 - 1.57)	<.001	
**Males**								
Person-years	212,872	1,887,855	1,089,713	770,205	275,282	115,367		
Colon cancer cases	176	1708	1064	737	274	118		0.155
MVHR (95% CI)[b]	0.88 (0.75 – 1.03)	0.91 (0.85 – 0.99)	Ref.	1.07 (0.98 – 1.18)	1.24 (1.09 – 1.42)	1.53 (1.26 – 1.85)	<.001	
**Females**								
Person-years	282,980	2,198,959	1,208,738	872,131	400,513	290,506		
Colon cancer cases	173	1303	814	643	293	168		
MVHR (95% CI)[b]	0.98 (0.83 – 1.16)	0.98 (0.90 – 1.08)	Ref.	1.10 (0.99 – 1.22)	1.19 (1.04 – 1.37)	1.34 (1.13 – 1.58)	<.001	
**Topography of tumor: rectal cancer**								
**Total**								
Person-years	495,854	4,088,442	2,300,577	1,644,833	676,912	406,095		
Rectal cancer cases	187	1694	938	676	276	119		
MVHR (95% CI)[a]	0.95 (0.81 - 1.12)	1.02 (0.94 - 1.11)	Ref.	1.07 (0.97 - 1.19)	1.15 (1.01 - 1.32)	1.18 (0.97 - 1.43)	0.017	
**Males**								
Person-years	213,124	1,889,065	1,091,024	771,549	275,983	115,480		
Rectal cancer cases	106	1076	581	409	148	55		0.574
MVHR (95% CI)[b]	0.91 (0.73 - 1.12)	1.02 (0.92 - 1.12)	Ref.	1.09 (0.96 - 1.24)	1.20 (1.00 - 1.44)	1.35 (1.02 - 1.79)	0.005	
**Females**								
Person-years	282,731	2,199,377	1,209,554	873,285	400,930	290,616		
Rectal cancer cases	81	618	357	267	128	64		
MVHR (95% CI)[b]	1.01 (0.79 - 1.29)	1.03 (0.90 - 1.17)	Ref.	1.04 (0.89 - 1.23)	1.10 (0.90 - 1.35)	1.05 (0.80 - 1.37)	0.580	

BMI: body mass index, CRC: colorectal cancer

[a]Model adjusted for age at baseline, sex, diabetes at baseline, current smoking, ever alcohol use, and enrollment period

[b]Model adjusted for age at baseline, diabetes at baseline, current smoking, ever alcohol use, and enrollment period

[c]P value was obtained form the interaction test between BMI and sex after model adjustment

© 2024 Paragomi P et al. *JAMA Network Open*.

eTable 6. Associations Between BMI and CRC Subtypes Incidence by Sex Among Those With Complete Data on Covariates

	BMI (kg/m^2)							
	**< 18.5**	**18.5 - 23**	**23 - 25**	**25 - 27.5**	**27.5 - 30**	**≥ 30**	***P for trend***	***P***
**Topography: colon cancer**								
**Total**								
Person-years	404,076	2,983,595	1,652,235	1,192,792	515,194	342,133		
Colon cancer cases	263	1935	1174	871	348	196		
MVHR (95% CI)[a]	0.92 (0.80 - 1.05)	0.95 (0.88 - 1.02)	Ref.	1.10 (1.01 - 1.20)	1.17 (1.04 - 1.32)	1.39 (1.19 - 1.62)	<.001	
**Males**								
Person-years	174,895	1,365,933	753,719	545,067	203,307	91,548		
Colon cancer cases	130	1076	628	448	151	75		0.153
MVHR (95% CI)[b]	0.85 (0.71 - 1.04)	0.91 (0.82 - 1.01)	Ref.	1.12 (0.99 - 1.26)	1.16 (0.97 - 1.39)	1.55 (1.22 - 1.98)	<.001	
**Females**								
Person-years	229,182	1,617,662	898,517	647,726	311,726	250,586		
Colon cancer cases	133	859	546	423	197	121		
MVHR (95% CI)[b]	1.01 (0.83 - 1.23)	1.01 (0.90 - 1.12)	Ref.	1.09 (0.96 - 1.24)	1.20 (1.02 - 1.42)	1.36 (1.11 - 1.66)	0.001	
**Topography of tumor: rectal cancer**								
**Total**								
Person-years	403,896	2,982,860	1,652,403	1,193,828	515,616	342,211		
Rectal cancer cases	153	1209	647	436	203	86		
MVHR (95% CI)[a]	0.95 (0.79 - 1.13)	1.02 (0.93 - 1.12)	Ref.	1.01 (0.90 - 1.15)	1.18 (1.01 - 1.39)	1.13 (0.89 - 1.41)	0.099	
**Males**								
Person-years	175,050	1,365,806	753,799	545,654	203,605	91,586		
Rectal cancer cases	89	761	387	250	102	35		0.883
MVHR (95% CI)[b]	0.92 (0.73 - 1.16)	1.00 (0.88 - 1.13)	Ref.	1.00 (0.86 - 1.18)	1.21 (0.97 - 1.50)	1.24 (0.87 - 1.75)	0.084	
**Females**								
	228,846	1,617,054	898,604	648,175	312,012	250,626		
Rectal cancer cases	64	448	260	186	101	51		
MVHR (95% CI)[b]	1.00 (0.76 - 1.32)	1.05 (0.90 - 1.23)	Ref.	1.00 (0.83 - 1.21)	1.14 (0.90 - 1.44)	1.03 (0.76 - 1.39)	0.844	

BMI: body mass index, CRC: colorectal cancer

[a]Model adjusted for age at baseline, sex, education, marital status, diabetes at baseline, current smoking, ever alcohol use, and enrollment period

[b]Model adjusted for age at baseline, education, marital status, diabetes at baseline, current smoking, ever alcohol use, and enrollment period

[c]P value was obtained form the interaction test between BMI and sex after model adjustment

© 2024 Paragomi P et al. *JAMA Network Open*.

eTable 7. Associations Between BMI and CRC Histologic Subtypes Mortality by Sex

	BMI (kg/m^2)							
	**< 18.5**	**18.5 - 23**	**23 - 25**	**25 - 27.5**	**27.5 - 30**	**≥ 30**	***P for trend***	$P_{heterogeneity}$[c]
**Topography: colon cancer**								
**Total**								
Person-years	511,436	4,267,499	2,411,707	1,741,818	720,981	427,652		
Colon cancer deaths	170	1285	698	471	229	136		
MVHR (95% CI)[a]	0.86 (0.73 - 1.03)	0.99 (0.90 - 1.09)	Ref.	1.00 (0.89 - 1.13)	1.25 (1.07 - 1.45)	1.53 (1.27 - 1.84)	<.001	
**Males**								
Person-years	219,565	1,976,897	1,148,456	822,568	296,468	123,508		
Colon cancer deaths	93	730	381	250	110	59		0.072
MVHR (95% CI)[b]	0.90 (0.72 - 1.13)	0.96 (0.85 - 1.09)	Ref.	1.02 (0.87 - 1.20)	1.34 (1.08 - 1.65)	1.99 (1.51 - 2.63)	<.001	
**Females**								
Person-years	291,872	2,290,603	1,263,252	919,250	424,514	304,144		
Colon cancer deaths	77	555	317	221	119	77		
MVHR (95% CI)[b]	0.82 (0.64 - 1.06)	1.03 (0.89 - 1.18)	Ref.	0.98 (0.83 - 1.17)	1.17 (0.95 - 1.45)	1.29 (1.00 - 1.67)	0.021	
**Topography of tumor: rectal cancer**								
**Total**								
Person-years	511,436	4,267,499	2,411,707	1,741,818	720,981	427,652		
Rectal cancer deaths	112	688	373	238	103	47		
MVHR (95% CI)[a]	1.16 (0.94 - 1.44)	0.99 (0.87 - 1.12)	Ref.	0.93 (0.79 - 1.09)	1.06 (0.85 - 1.32)	1.08 (0.79 - 1.46)	0.588	
**Males**								
Person-years	219,565	1,976,897	1,148,456	822,568	296,468	123,508		
Rectal cancer deaths	67	468	233	146	46	28		0.058
MVHR (95% CI)[b]	1.14 (0.87 - 1.51)	1.03 (0.88 - 1.20)	Ref.	0.96 (0.78 - 1.19)	0.92 (0.67 - 1.26)	1.63 (1.10 - 2.42)	0.911	
**Females**								
Person-years	291,872	2,290,603	1,263,252	919,250	424,514	304,144		
Rectal cancer deaths	45	220	140	92	57	19		
MVHR (95% CI)[b]	1.21 (0.86 - 1.70)	0.93 (0.75 - 1.15)	Ref.	0.87 (0.66 - 1.13)	1.17 (0.86 - 1.59)	0.67 (0.41 - 1.08)	0.317	

BMI: body mass index, CRC: colorectal cancer

[a]Model adjusted for age at baseline, sex, diabetes at baseline, current smoking, ever alcohol use, and enrollment period

[b]Model adjusted for age at baseline, diabetes at baseline, current smoking, ever alcohol use, and enrollment period

[c]P value was obtained form the interaction test between BMI and sex after model adjustment

© 2024 Paragomi P et al. *JAMA Network Open*.

eTable 8. Associations Between BMI and CRC Histologic Subtypes Mortality by Sex Among Those With Complete Data on Covariates

	BMI (kg/m²)							
	**< 18.5**	**18.5 - 23**	**23 - 25**	**25 - 27.5**	**27.5 - 30**	**≥ 30**	***P for trend***	$P_{heterogeneity}$[c]
**Topography: colon cancer**								
**Total**								
Person-years	419,181	3,156,570	1,760,317	1,288,383	558,787	363,401		
Colon cancer deaths	140	973	535	366	176	108		
MVHR (95% CI)[a]	0.82 (0.68 - 0.99)	0.96 (0.86 - 1.06)	Ref.	1.03 (0.90 - 1.17)	1.22 (1.03 - 1.45)	1.47 (1.19 - 1.81)	<.001	
**Males**								
Person-years	181,355	1,450,597	809,158	595,082	223,532	99,398		
Colon cancer deaths	76	553	274	198	79	45		0.124
MVHR (95% CI)[b]	0.87 (0.67 - 1.12)	0.95 (0.82 - 1.10)	Ref.	1.12 (0.93 - 1.35)	1.29 (1.00 - 1.66)	1.98 (1.44 - 2.73)	<.001	
**Females**								
Person-years	237,827	1,705,974	951,160	693,302	335,256	264,003		
Colon cancer deaths	64	420	261	168	97	63		
MVHR (95% CI)[b]	0.76 (0.58 - 1.01)	0.96 (0.82 - 1.13)	Ref.	0.93 (0.77 - 1.14)	1.16 (0.91 - 1.47)	1.21 (0.91 - 1.61)	0.015	
**Topography of tumor : rectal cancer**								
**Total**								
Person-years	419,181	3,156,570	1,760,317	1,288,383	558,787	363,401		
Rectal cancer deaths	95	507	268	173	85	39		
MVHR (95% CI)[a]	1.18 (0.93 - 1.50)	0.98 (0.84 - 1.14)	Ref.	0.93 (0.77 - 1.13)	1.15 (0.90 - 1.48)	1.11 (0.79 - 1.56)	0.999	
**Males**								
Person-years	181,355	1,450,597	809,158	595,082	223,532	99,398		
Rectal cancer deaths	60	340	164	100	37	21		0.318
MVHR (95% CI)[b]	1.20 (0.88 - 1.61)	0.98 (0.81 - 1.18)	Ref.	0.93 (0.73 - 1.20)	1.02 (0.71 - 1.46)	1.63 (1.03 - 2.58)	0.886	
**Females**								
Person-years	237,827	1,705,974	951,160	693,302	335,256	264,003		
Rectal cancer deaths	35	167	104	73	48	18		
MVHR (95% CI)[b]	1.19 (0.80 - 1.76)	0.99 (0.77 - 1.27)	Ref.	0.93 (0.68 - 1.25)	1.26 (0.89 - 1.78)	0.76 (0.46 - 1.27)	0.588	

BMI: body mass index, CRC: colorectal cancer

[a]Model adjusted for age at baseline, sex, education, marital status, diabetes at baseline, current smoking, ever alcohol use, and enrollment period

[b]Model adjusted for age at baseline, education, marital status, diabetes at baseline, current smoking, ever alcohol use, and enrollment period

[c]P value was obtained form the interaction test between BMI and sex after model adjustment

© 2024 Paragomi P et al. *JAMA Network Open*.

eTable 9. Associations Between BMI and CRC Mortality in Subgroups Among Those Having Complete Data on Covariates

	BMI (kg/m^2)							
**Subgroups**	**< 18.5**	**18.5 - 23**	**23 - 25**	**25 - 27.5**	**27.5 - 30**	**≥ 30**	***P for trend***	$P_{heterogeneity}$[e]
**Location**								
**Chinese origin (N=238097)**								
CRC deaths	116	760	473	332	179	88		
MVHR (95% CI)[a]	1.12 (0.91 - 1.37)	1.02 (0.91 - 1.15)	Ref.	1.01 (0.88 - 1.17)	1.28 (1.08 - 1.52)	1.36 (1.08 - 1.71)	0.040	
**South Korea (N=58375)**								
CRC deaths	3	39	14	22	6	1		
MVHR (95% CI)[a]	0.95 (0.27 - 3.34)	1.47 (0.79 - 2.72)	Ref.	1.97 (1.01 - 3.85)	1.41 (0.54 - 3.68)	0.66 (0.09 - 5.05)	0.732	0.234
**Japan (N=150691)**								
CRC deaths	112	667	306	174	63	30		
MVHR (95% CI)[a]	0.77 (0.62 - 0.96)	0.87 (0.76 - 1.00)	Ref.	0.92 (0.76 - 1.11)	1.01 (0.77 - 1.32)	1.12 (0.77 - 1.63)	0.012	
**Iran (N=49903)**								
CRC deaths	4	14	10	11	13	28		
MVHR (95% CI)[a]	1.19 (0.37 - 3.82)	0.85 (0.38 - 1.92)	Ref.	0.86 (0.36 - 2.02)	1.22 (0.53 - 2.80)	1.88 (0.90 - 3.95)	0.022	
**Diabetes Mellitus**								
**No diabetes (N=458648)**								
CRC deaths	212	1322	712	486	226	130		
MVHR (95% CI)[b]	0.94 (0.80 - 1.10)	0.95 (0.87 - 1.04)	Ref.	1.02 (0.91 - 1.15)	1.20 (1.03 - 1.40)	1.42 (1.18 - 1.72)	<.001	0.372
**Diabetes (N=28339)**								
CRC deaths	10	100	74	40	31	16		
MVHR (95% CI)[b]	1.06 (0.54 - 2.07)	1.06 (0.78 - 1.44)	Ref.	0.70 (0.48 - 1.04)	1.12 (0.73 - 1.71)	0.99 (0.57 - 1.73)	0.487	
**Smoking status**								
**Former or never smokers (N=357539)**								
CRC deaths	132	875	556	388	207	118		
MVHR (95% CI)[c]	0.95 (0.78 - 1.15)	0.95 (0.85 - 1.05)	Ref.	0.97 (0.85 - 1.11)	1.21 (1.03 - 1.42)	1.30 (1.06 - 1.60)	<.001	0.876
**Current smokers (N=134167)**								
CRC deaths	98	580	237	146	52	29		
MVHR (95% CI)[c]	0.96 (0.75 - 1.22)	1.00 (0.86 - 1.17)	Ref.	1.06 (0.86 - 1.30)	1.11 (0.82 - 1.51)	1.60 (1.08 - 2.35)	0.073	
**Alcohol use status**								
**Never alcohol users (N=326524)**								
CRC deaths	141	851	496	358	196	112		
MVHR (95% CI)[d]	0.98 (0.81 - 1.19)	0.98 (0.88 - 1.10)	Ref.	1.02 (0.89 - 1.17)	1.28 (1.08 - 1.51)	1.30 (1.06 - 1.61)	0.001	0.672
**Ever alcohol users (N=139622)**								
CRC deaths	78	514	263	158	62	34		
MVHR (95% CI)[d]	0.90 (0.69 - 1.16)	0.92 (0.79 - 1.07)	Ref.	0.94 (0.77 - 1.15)	1.08 (0.82 - 1.42)	1.67 (1.16 - 2.39)	0.019	

BMI: body mass index, CRC: colorectal cancer

[a]Model adjusted for age at baseline, sex, education, marital status, diabetes at baseline, current smoking, and ever alcohol use

[b]Model adjusted for age at baseline, sex, education, marital status, current smoking, and ever alcohol use

[c]Model adjusted for age at baseline, sex, education, marital status, diabetes at baseline, and ever alcohol use

[d]Model adjusted for age at baseline, sex, education, marital status, diabetes at baseline, and current smoking

[e]P value was obtained form the interaction test between BMI and subgroup after model adjustment

© 2024 Paragomi P et al. *JAMA Network Open*.

eTable 10. Associations Between BMI and CRC Incidence in Subgroups Among Those With Complete Data on Covariates

	BMI (kg/m²)							
**Subgroups**	**< 18.5**	**18.5 - 23**	**23 - 25**	**25 - 27.5**	**27.5 - 30**	**≥ 30**	***P for trend***	***P*$_{heterogeneity}$[e]**
**Location**								
**Chinese origin (N=209154)**								
Person-years	158,344	1,290,323	817,987	573,781	234,600	109,656		
CRC cases	187	1450	911	679	321	138		
MVHR (95% CI)[a]	0.96 (0.82 - 1.12)	1.03 (0.94 - 1.12)	Ref.	1.12 (1.01 - 1.24)	1.26 (1.11 - 1.43)	1.15 (0.96 - 1.38)	<.001	
**South Korea (N=58201)**								
Person-years	20,672	244,086	161,282	133,997	49,616	19,190		
CRC cases	7	106	77	79	28	11		
MVHR (95% CI)[a]	0.61 (0.28 - 1.33)	0.88 (0.66 - 1.19)	Ref.	1.23 (0.90 - 1.69)	1.14 (0.74 - 1.75)	1.26 (0.67 - 2.37)	0.013	0.415
**Japan (N=150099)**								
Person-years	196,350	1,304,703	582,589	368,524	130,669	51,406		
CRC cases	247	1821	938	625	207	97		
MVHR (95% CI)[a]	0.90 (0.78 - 1.04)	0.94 (0.87 - 1.02)	Ref.	1.05 (0.94 - 1.16)	1.08 (0.92 - 1.25)	1.39 (1.13 - 1.72)	<.001	
**Iran (N=49884)**								
Person-years	27,457	133,652	85,439	113,490	99,298	161,491		
CRC cases	3	21	14	23	21	45		
MVHR (95% CI)[a]	0.71 (0.20 - 2.49)	0.96 (0.49 - 1.90)	Ref.	1.25 (0.64 - 2.42)	1.35 (0.68 - 2.66)	2.10 (1.14 - 3.87)	<.001	
**Diabetes Mellitus**								
**No diabetes (N=429779)**								
Person-years	362,727	2,747,538	1,523,170	1,094,430	467,127	305,046		
CRC cases	407	3093	1741	1248	510	250		
MVHR (95% CI)[b]	0.92 (0.83 - 1.03)	0.98 (0.92 - 1.04)	Ref.	1.08 (1.01 - 1.16)	1.20 (1.08 - 1.32)	1.31 (1.14 - 1.50)	<.001	0.697
**Diabetes (N=27486)**								
Person-years	10,962	109,586	87,263	72,979	39,438	31,935		
CRC cases	13	196	166	130	61	35		
MVHR (95% CI)[b]	0.64 (0.36 - 1.14)	0.90 (0.73 - 1.11)	Ref.	1.09 (0.86 - 1.38)	1.06 (0.79 - 1.43)	1.14 (0.78 - 1.65)	0.029	
**Smoking status**								
**Former or never smokers (N=335282)**								
Person-years	258,436	2,032,598	1,205,221	900,322	411,809	298,770		
CRC cases	247	2080	1301	1006	443	243		
MVHR (95% CI)[c]	0.92 (0.80 - 1.06)	1.01 (0.94 - 1.08)	Ref.	1.10 (1.02 - 1.20)	1.24 (1.11 - 1.38)	1.37 (1.19 - 1.57)	<.001	0.451
**Current smokers (N=126703)**								
Person-years	134,929	897,844	424,140	278,423	98,907	40,728		
CRC cases	186	1270	622	388	130	47		
MVHR (95% CI)[c]	0.88 (0.75 - 1.04)	0.90 (0.82 - 1.00)	Ref.	1.07 (0.94 - 1.21)	1.05 (0.87 - 1.27)	1.11 (0.83 - 1.50)	<.001	
**Alcohol use status**								
**Never alcohol users (N=300333)**								
Person-years	262,354	1,834,609	1,054,852	795,678	376,947	292,590		
CRC cases	245	1829	1044	807	387	206		
MVHR (95% CI)[d]	0.97 (0.84 - 1.12)	1.04 (0.96 - 1.12)	Ref.	1.11 (1.01 - 1.22)	1.26 (1.12 - 1.42)	1.30 (1.11 - 1.51)	<.001	0.282
**Ever alcohol users (N=136181)**								
CRC cases	165	1252	768	511	172	76		
MVHR (95% CI)[d]	0.92 (0.78 - 1.10)	0.89 (0.81 - 0.98)	Ref.	1.04 (0.93 - 1.16)	1.08 (0.92 - 1.28)	1.33 (1.05 - 1.68)	<.001	

BMI: body mass index, CRC: colorectal cancer
[a]Model adjusted for age at baseline, sex, education, marital status, diabetes at baseline, current smoking, ever alcohol use, and enrollment period
[b]Model adjusted for age at baseline, sex, education, marital status, current smoking, ever alcohol use, and enrollment period
[c]Model adjusted for age at baseline, sex, education, marital status, diabetes at baseline, ever alcohol use, and enrollment period
[d]Model adjusted for age at baseline, sex, education, marital status, diabetes at baseline, current smoking, and enrollment period
[e]P value was obtained form the interaction test between BMI and subgroup after model adjustment

© 2024 Paragomi P et al. *JAMA Network Open*.

eTable 11. Associations Between BMI and CRC Mortality in Subgroups

	BMI (kg/m^2)							
**Subgroups**	**< 18.5**	**18.5 - 23**	**23 - 25**	**25 - 27.5**	**27.5 - 30**	**≥ 30**	***P* for trend**	***P*$_{heterogeneity}$[e]**
**Location**								
**Chinese origin (N=246222)**								
	179,987	1,508,446	952,588	690,113	285,038	133,212		
CRC deaths	116	775	483	339	183	90		
MVHR (95% CI)[a]	1.10 (0.90 - 1.36)	1.02 (0.91 - 1.15)	Ref.	1.01 (0.88 - 1.16)	1.29 (1.08 - 1.53)	1.38 (1.10 - 1.73)	0.029	
**South Korea (N=58375)**								
Person-years	20,761	245,236	162,054	134,562	49,841	19,301		
CRC deaths	3	39	14	22	6	1		
MVHR (95% CI)[a]	0.98 (0.28 - 3.46)	1.49 (0.81 - 2.76)	Ref.	1.97 (1.01 - 3.86)	1.38 (0.53 - 3.59)	0.66 (0.09 - 5.00)	0.809	0.416
**Japan (N=295580)**								
Person-years	283,244	2,379,774	1,211,435	803,348	286,465	113,051		
CRC deaths	159	1145	564	337	130	64		
MVHR (95% CI)[a]	0.88 (0.74 - 1.05)	0.96 (0.86 - 1.06)	Ref.	0.92 (0.81 - 1.05)	1.04 (0.86 - 1.26)	1.27 (0.98 - 1.65)	0.074	
**Iran (N=50018)**								
Person-years	27,545	134,045	85,631	113,798	99,638	162,088		
CRC deaths	4	14	10	11	13	28		
MVHR (95% CI)[a]	1.16 (0.36 - 3.73)	0.83 (0.37 - 1.88)	Ref.	0.87 (0.37 - 2.04)	1.26 (0.55 - 2.88)	1.92 (0.92 - 4.02)	0.015	
**Diabetes Mellitus**								
**No diabetes (N=604076)**								
Person-years	467,942	3,998,116	2,255,679	1,620,256	662,722	385,271		
CRC deaths	257	1782	966	638	289	165		
MVHR (95% CI)[b]	0.96 (0.84 - 1.11)	0.98 (0.90 - 1.06)	Ref.	0.98 (0.89 - 1.09)	1.17 (1.02 - 1.33)	1.45 (1.23 - 1.71)	<.001	0.647
**Diabetes (N=36040)**								
CRC deaths	12	133	88	58	39	17		
MVHR (95% CI)[b]	1.06 (0.58 - 1.95)	1.13 (0.86 - 1.48)	Ref.	0.87 (0.62 - 1.21)	1.26 (0.86 - 1.84)	1.01 (0.59 - 1.72)	0.670	
**Smoking status**								
**Former or never smokers (N=463000)**								
Person-years	332,149	2,926,863	1,755,437	1,311,477	570,031	366,333		
CRC deaths	162	1167	721	502	259	139		
MVHR (95% CI)[c]	0.99 (0.84 - 1.18)	0.99 (0.90 - 1.08)	Ref.	0.97 (0.86 - 1.08)	1.21 (1.05 - 1.40)	1.30 (1.08 - 1.57)	0.004	0.633
**Current smokers (N=181835)**								
Person-years	169,871	1,298,320	638,399	419,375	147,487	59,066		
CRC deaths	115	781	340	202	71	44		
MVHR (95% CI)[c]	0.95 (0.76 - 1.17)	1.00 (0.88 - 1.13)	Ref.	1.01 (0.85 - 1.20)	1.07 (0.83 - 1.38)	1.77 (1.29 - 2.42)	0.032	
**Alcohol use status**								
**Never alcohol users (N=421155)**								
Person-years	337,754	2,721,126	1,583,831	1,191,780	530,718	358,808		
CRC deaths	172	1149	648	467	242	141		
MVHR (95% CI)[d]	1.04 (0.88 - 1.23)	1.02 (0.93 - 1.13)	Ref.	1.01 (0.89 - 1.14)	1.25 (1.08 - 1.45)	1.40 (1.16 - 1.68)	0.003	0.830
**Ever alcohol users (N=198120)**								
CRC deaths	94	709	379	219	87	41		
MVHR (95% CI)[d]	0.87 (0.69 - 1.10)	0.93 (0.82 - 1.05)	Ref.	0.92 (0.78 - 1.09)	1.08 (0.85 - 1.36)	1.44 (1.04 - 1.99)	0.027	

BMI: body mass index, CRC: colorectal cancer

[a]Model adjusted for age at baseline, sex, diabetes at baseline, current smoking, ever alcohol use, and enrollment period

[b]Model adjusted for age at baseline, sex, current smoking, ever alcohol use, and enrollment period

[c]Model adjusted for age at baseline, sex, diabetes at baseline, ever alcohol use, and enrollment period

[d]Model adjusted for age at baseline, sex, diabetes at baseline, current smoking, and enrollment period

[e]P value was obtained form the interaction test between BMI and subgroup after model adjustment

© 2024 Paragomi P et al. *JAMA Network Open*.

eTable 12. Associations Between BMI and CRC Incidence by Sex in Subgroups

	BMI (kg/m²)						*P* for trend	$P_{heterogeneity}$[i]
	< 18.5	18.5 - 23	23 - 25	25 - 27.5	27.5 - 30	≥ 30		
**Chinese origin (N=217190)**								
**Total**								
Person-years	162,982	1,327,468	842,068	593,872	241,452	112,089		
CRC cases	190	1476	940	697	330	141		
MVHR (95% CI)[a]	0.95 (0.81 - 1.11)	1.02 (0.94 - 1.10)	Ref.	1.11 (1.00 - 1.22)	1.26 (1.11 - 1.42)	1.15 (0.96 - 1.37)	<.001	
**Males**								
Person-years	90,346	645,498	361,762	264,847	90,433	39,616		
CRC cases	126	889	482	350	147	54		0.457
MVHR (95% CI)[b]	0.95 (0.78 - 1.16)	0.98 (0.88 - 1.09)	Ref.	1.08 (0.94 - 1.24)	1.28 (1.07 - 1.55)	1.40 (1.06 - 1.85)	<.001	
**Females**								
Person-years	72,636	681,971	480,307	329,025	151,019	81,473		
CRC cases	64	587	458	347	183	87		
MVHR (95% CI)[b]	0.95 (0.73 - 1.23)	1.07 (0.94 - 1.21)	Ref.	1.14 (0.99 - 1.32)	1.23 (1.04 - 1.47)	1.04 (0.83 - 1.31)	0.140	
**South Korea (N=58201)**								
**Total**								
Person-years	20,672	244,086	161,282	133,997	49,616	19,190		
CRC cases	7	106	77	79	28	11		
MVHR (95% CI)[a]	0.61 (0.28 - 1.33)	0.89 (0.66 - 1.19)	Ref.	1.24 (0.90 - 1.69)	1.14 (0.74 - 1.76)	1.28 (0.68 - 2.41)	0.012	
**Males**								
Person-years	6,899	94,463	81,624	73,172	25,170	7,771		
CRC cases	5	58	39	45	13	4		0.909
MVHR (95% CI)[b]	0.88 (0.34 - 2.25)	0.97 (0.64 - 1.46)	Ref.	1.46 (0.95 - 2.24)	1.25 (0.67 - 2.35)	1.47 (0.53 - 4.13)	0.075	
**Females**								
Person-years	13,774	149,623	79,658	60,826	24,446	11,420		
CRC cases	2	48	38	34	15	7		
MVHR (95% CI)[b]	0.37 (0.09 - 1.52)	0.78 (0.51 - 1.20)	Ref.	1.03 (0.65 - 1.64)	1.03 (0.56 - 1.87)	1.13 (0.51 - 2.54)	0.073	
**Japan (N=294592)**								
**Total**								
Person-years	277,522	2,296,133	1,161,778	767,807	273,062	108,175		
CRC cases	364	3356	1906	1357	491	217		
MVHR (95% CI)[a]	0.92 (0.82 - 1.03)	0.95 (0.90 - 1.01)	Ref.	1.08 (1.00 - 1.15)	1.15 (1.04 - 1.27)	1.37 (1.19 - 1.58)	<.001	
**Males**								
Person-years	98,471	1,030,357	578,036	363,763	115,182	37,061		
CRC cases	164	1965	1195	808	271	104		0.233
MVHR (95% CI)[b]	0.85 (0.72 - 1.00)	0.94 (0.87 - 1.01)	Ref.	1.09 (1.00 - 1.19)	1.21 (1.06 - 1.38)	1.46 (1.19 - 1.78)	<.001	
**Females**								
Person-years	179,052	1,265,777	583,742	404,044	157,881	71,114		
CRC cases	200	1391	711	549	220	113		
MVHR (95% CI)[b]	0.99 (0.84 - 1.16)	0.98 (0.90 - 1.08)	Ref.	1.05 (0.94 - 1.18)	1.10 (0.95 - 1.29)	1.35 (1.11 - 1.65)	0.004	
**Iran (N=49998)**								
**Total**								
Person-years	27,523	133,982	85,559	113,671	99,550	161,935		
CRC cases	3	21	14	23	21	45		
MVHR (95% CI)[a]	0.65 (0.19 - 2.28)	0.90 (0.46 - 1.78)	Ref.	1.28 (0.66 - 2.48)	1.41 (0.72 - 2.79)	2.19 (1.19 - 4.04)	<.001	
**Males**								
Person-years	13,867	74,165	41,318	50,594	38,920	38,131		
CRC cases	2	17	9	16	12	17		0.976
MVHR (95% CI)[b]	0.67 (0.14 - 3.12)	1.05 (0.47 - 2.37)	Ref.	1.45 (0.64 - 3.29)	1.44 (0.61 - 3.44)	2.15 (0.95 - 4.85)	0.019	
**Females**								
Person-years	13,656	59,818	44,241	63,077	60,630	123,805		
CRC cases	1	4	5	7	9	28		
MVHR (95% CI)[b]	0.61 (0.07 - 5.26)	0.58 (0.16 - 2.17)	Ref.	0.99 (0.31 - 3.12)	1.34 (0.45 - 4.01)	2.07 (0.80 - 5.37)	0.003	
**Diabetes (N=35153)**								
**Total**								
Person-years	14,157	148,269	113,998	94,539	48,076	34,416		
CRC cases	16	282	221	181	82	47		
MVHR (95% CI)[c]	0.58 (0.35 - 0.97)	0.96 (0.80 - 1.14)	Ref.	1.14 (0.94 - 1.39)	1.12 (0.87 - 1.45)	1.25 (0.91 - 1.73)	0.003	
**Males**								
Person-years	7,643	87,097	63,332	49,967	21,579	9,906		

© 2024 Paragomi P et al. *JAMA Network Open*.

	BMI (kg/m²)							
	< 18.5	18.5 - 23	23 - 25	25 - 27.5	27.5 - 30	≥ 30	*P for trend*	$P_{heterogeneity}$[i]
CRC cases	13	198	148	116	51	23		0.538
MVHR (95% CI)[d]	0.75 (0.42 - 1.32)	0.99 (0.80 - 1.23)	Ref.	1.16 (0.91 - 1.48)	1.23 (0.89 - 1.70)	1.48 (0.95 - 2.31)	0.012	
**Females**								
Person-years	6,514	61,173	50,667	44,573	26,498	26,510		
CRC cases	3	84	73	65	31	24		
MVHR (95% CI)[d]	0.27 (0.09 - 0.88)	0.88 (0.63 - 1.20)	Ref.	1.07 (0.76 - 1.50)	0.91 (0.60 - 1.40)	1.01 (0.63 - 1.62)	0.132	
**No diabetes (N=574755)**								
**Total**								
Person-years	445,408	3,737,759	2,099,824	1,492,426	607,986	360,211		
CRC cases	524	4568	2683	1947	782	361		
MVHR (95% CI)[c]	0.93 (0.84 - 1.02)	0.97 (0.92 - 1.01)	Ref.	1.08 (1.02 - 1.15)	1.20 (1.11 - 1.30)	1.31 (1.17 - 1.47)	<.001	
**Males**								
Person-years	199,037	1,744,101	994,581	699,209	247,125	103,270		
CRC cases	281	2712	1574	1102	389	156		0.199
MVHR (95% CI)[d]	0.88 (0.78 - 1.01)	0.94 (0.89 - 1.01)	Ref.	1.09 (1.01 - 1.18)	1.22 (1.09 - 1.37)	1.44 (1.22 - 1.70)	<.001	
**Females**								
Person-years	246,372	1,993,659	1,105,244	793,218	360,861	256,942		
CRC cases	243	1856	1109	845	393	205		
MVHR (95% CI)[d]	0.99 (0.86 - 1.14)	1.00 (0.93 - 1.08)	Ref.	1.07 (0.98 - 1.17)	1.19 (1.06 - 1.34)	1.25 (1.07 - 1.45)	<.001	
**Current smokers (N=174204)**								
**Total**								
Person-years	164,141	1,223,424	595,647	386,647	134,426	53,798		
CRC cases	235	1889	950	626	204	82		
MVHR (95% CI)[e]	0.92 (0.79 - 1.06)	0.94 (0.87 - 1.01)	Ref.	1.11 (1.00 - 1.23)	1.10 (0.94 - 1.28)	1.29 (1.03 - 1.62)	<.001	
**Males**								
Person-years	132,623	1,077,715	534,133	346,203	116,044	42,021		
CRC cases	196	1725	871	584	184	71		0.081
MVHR (95% CI)[f]	0.92 (0.79 - 1.08)	0.94 (0.87 - 1.02)	Ref.	1.14 (1.02 - 1.26)	1.19 (1.01 - 1.39)	1.40 (1.10 - 1.78)	<.001	
**Females**								
Person-years	31,518	145,710	61,514	40,005	18,383	11,777		
CRC cases	39	164	79	42	20	11		
MVHR (95% CI)[f]	0.82 (0.55 - 1.23)	0.89 (0.68 - 1.18)	Ref.	0.85 (0.58 - 1.24)	0.64 (0.38 - 1.06)	0.83 (0.44 - 1.57)	0.586	
**Former or never smokers (N=440424)**								
**Total**								
Person-years	315,100	2,735,922	1,637,104	1,212,093	525,788	345,346		
CRC cases	318	3022	1970	1518	662	331		
MVHR (95% CI)[e]	0.91 (0.81 - 1.03)	0.98 (0.93 - 1.04)	Ref.	1.09 (1.02 - 1.16)	1.23 (1.13 - 1.35)	1.34 (1.19 - 1.51)	<.001	
**Males**								
Person-years	76,238	761,636	525,904	404,612	153,096	71,381		
CRC cases	100	1199	850	634	258	108		0.156
MVHR (95% CI)[f]	0.82 (0.66 - 1.01)	0.96 (0.87 - 1.04)	Ref.	1.07 (0.96 - 1.18)	1.27 (1.11 - 1.47)	1.51 (1.23 - 1.85)	<.001	
**Females**								
Person-years	238,863	1,974,287	1,111,200	807,481	372,693	273,965		
CRC cases	218	1823	1120	884	404	223		
MVHR (95% CI)[f]	0.97 (0.84 - 1.13)	1.00 (0.93 - 1.08)	Ref.	1.09 (1.00 - 1.20)	1.20 (1.07 - 1.35)	1.27 (1.10 - 1.47)	<.001	
**Ever alcohol users (N=194493)**								
**Total**								
Person-years	139,377	1,260,917	697,960	473,893	166,196	60,699		
CRC cases	202	1819	1127	740	272	106		
MVHR (95% CI)[g]	0.88 (0.76 - 1.03)	0.90 (0.84 - 0.97)	Ref.	1.03 (0.94 - 1.13)	1.16 (1.01 - 1.32)	1.28 (1.04 - 1.56)	<.001	
**Males**								
Person-years	90,880	911,135	539,579	371,773	125,591	41,429		
CRC cases	156	1514	961	645	224	85		0.149
MVHR (95% CI)[h]	0.89 (0.75 - 1.06)	0.90 (0.83 - 0.97)	Ref.	1.07 (0.96 - 1.18)	1.16 (1.00 - 1.34)	1.39 (1.12 - 1.74)	<.001	
**Females**								
Person-years	48,497	349,782	158,382	102,121	40,605	19,271		
CRC cases	46	305	166	95	48	21		
MVHR (95% CI)[h]	0.78 (0.56 - 1.09)	0.93 (0.77 - 1.13)	Ref.	0.81 (0.62 - 1.04)	1.09 (0.79 - 1.51)	0.96 (0.60 - 1.51)	0.527	

© 2024 Paragomi P et al. *JAMA Network Open*.

	BMI (kg/m²)							
	**< 18.5**	**18.5 - 23**	**23 - 25**	**25 - 27.5**	**27.5 - 30**	**≥ 30**	***P for trend***	$P_{heterogeneity}$ [i]
**Never alcohol users (N=394664)**								
**Total**								
Person-years	318,632	2,513,180	1,459,410	1,086,793	483,506	336,555		
CRC cases	328	2823	1682	1328	580	299		
MVHR (95% CI)[g]	0.98 (0.87 - 1.11)	1.01 (0.95 - 1.07)	Ref.	1.12 (1.04 - 1.20)	1.23 (1.12 - 1.35)	1.34 (1.18 - 1.51)	<.001	
**Males**								
Person-years	108,422	838,870	476,804	355,930	137,670	70,630		
CRC cases	125	1244	688	525	207	90		0.306
MVHR (95% CI)[h]	0.89 (0.73 - 1.08)	1.01 (0.92 - 1.11)	Ref.	1.12 (1.00 - 1.26)	1.33 (1.13 - 1.55)	1.54 (1.23 - 1.92)	<.001	
**Females**								
Person-years	210,211	1,674,311	982,606	730,863	345,837	265,925		
CRC cases	203	1579	994	803	373	209		
MVHR (95% CI)[h]	1.05 (0.90 - 1.23)	1.02 (0.94 - 1.10)	Ref.	1.12 (1.02 - 1.23)	1.19 (1.05 - 1.34)	1.27 (1.09 - 1.48)	<.001	

BMI: body mass index, CRC: colorectal cancer
[a]Model adjusted for age at baseline, sex, diabetes at baseline, current smoking, ever alcohol use, and enrollment period
[b]Model adjusted for age at baseline, diabetes at baseline, current smoking, ever alcohol use, and enrollment period
[c]Model adjusted for age at baseline, sex, current smoking, ever alcohol use, and enrollment period
[d]Model adjusted for age at baseline, current smoking, ever alcohol use, and enrollment period
[e]Model adjusted for age at baseline, sex, diabetes at baseline, ever alcohol use, and enrollment period
[f]Model adjusted for age at baseline, diabetes at baseline, ever alcohol use, and enrollment period
[g]Model adjusted for age at baseline, sex, diabetes at baseline, current smoking, and enrollment period
[h]Model adjusted for age at baseline, diabetes at baseline, current smoking, and enrollment period
[i]P value was obtained form the interaction test between BMI and sex after model adjustment

© 2024 Paragomi P et al. *JAMA Network Open*.

eTable 13. Associations Between BMI and CRC Incidence by Sex in Subgroups Among Those With Complete Data on Covariates

	BMI (kg/m^2)							
	< 18.5	18.5 - 23	23 - 25	25 - 27.5	27.5 - 30	≥ 30	*P for trend*	$P_{heterogeneity}$[i]
**Chinese origin (N=209154)**								
**Total**								
Person-years	158,344	1,290,323	817,987	573,781	234,600	109,656		
CRC cases	187	1450	911	679	321	138		
MVHR (95% CI)[a]	0.96 (0.82 - 1.12)	1.03 (0.94 - 1.12)	Ref.	1.12 (1.01 - 1.24)	1.26 (1.11 - 1.43)	1.15 (0.96 - 1.38)	<.001	
**Males**								
Person-years	85,708	608,352	337,681	244,756	83,581	28,184		
CRC cases	123	863	453	332	138	51		0.534
MVHR (95% CI)[b]	0.97 (0.79 - 1.19)	1.00 (0.89 - 1.12)	Ref.	1.10 (0.95 - 1.27)	1.30 (1.07 - 1.57)	1.42 (1.07 - 1.91)	0.001	
**Females**								
Person-years	72,636	681,971	480,307	329,025	151,-19	81,473		
CRC cases	64	587	458	347	183	87		
MVHR (95% CI)[b]	0.94 (0.73 - 1.23)	1.07 (0.94 - 1.21)	Ref.	1.14 (0.99 - 1.32)	1.23 (1.04 - 1.47)	1.04 (0.83 - 1.31)	0.140	
**South Korea (N=58201)**								
**Total**								
Person-years	20,672	244,086	161,282	133,997	49,616	19,190		
CRC cases	7	106	77	79	28	11		
MVHR (95% CI)[a]	0.61 (0.28 - 1.33)	0.88 (0.66 - 1.19)	Ref.	1.23 (0.90 - 1.69)	1.14 (0.74 - 1.75)	1.26 (0.67 - 2.37)	0.013	
**Males**								
Person-years	6,899	94,463	81,624	73,172	25,170	7,771		
CRC cases	5	58	39	45	13	4		0.832
MVHR (95% CI)[b]	0.90 (0.35 - 2.33)	0.97 (0.64 - 1.46)	Ref.	1.45 (0.94 - 2.23)	1.25 (0.67 - 2.35)	1.45 (0.52 - 4.08)	0.082	
**Females**								
Person-years	13,774	149,623	79,658	60,826	24,446	11,420		
CRC cases	2	48	38	34	15	7		
MVHR (95% CI)[b]	0.35 (0.08 - 1.47)	0.78 (0.51 - 1.20)	Ref.	1.02 (0.64 - 1.62)	1.01 (0.55 - 1.84)	1.11 (0.49 - 2.48)	0.083	
**Japan (N=150099)**								
**Total**								
Person-years	196,350	1,304,703	582,589	368,524	130,669	51,406		
CRC cases	247	1821	938	625	207	97		
MVHR (95% CI)[a]	0.90 (0.78 - 1.04)	0.94 (0.87 - 1.02)	Ref.	1.05 (0.94 - 1.16)	1.08 (0.92 - 1.25)	1.39 (1.13 - 1.72)	<.001	
**Males**								
Person-years	67,127	579,379	289,058	174,070	54,970	17,290		
CRC cases	104	1044	592	377	110	44		0.291
MVHR (95% CI)[b]	0.81 (0.65 - 0.99)	0.90 (0.82 - 1.00)	Ref.	1.08 (0.95 - 1.23)	1.08 (0.88 - 1.33)	1.37 (1.01 - 1.87)	<.001	
**Females**								
Person-years	129,223	725,325	293,532	194,454	75,700	34,117		
CRC cases	143	777	346	248	97	53		
MVHR (95% CI)[b]	1.01 (0.82 - 1.23)	1.01 (0.89 - 1.15)	Ref.	0.99 (0.84 - 1.17)	1.08 (0.86 - 1.35)	1.53 (1.14 - 2.04)	0.143	
**Iran (N=49884)**								
**Total**								
Person-years	27,457	133,652	85,439	113,490	99,298	161,491		
CRC cases	3	21	14	23	21	45		
MVHR (95% CI)[a]	0.71 (0.20 - 2.49)	0.96 (0.49 - 1.90)	Ref.	1.25 (0.64 - 2.42)	1.35 (0.68 - 2.66)	2.10 (1.14 - 3.87)	<.001	
**Males**								
Person-years	13,867	74,082	41,305	50,552	38,889	38,090		
CRC cases	2	17	9	16	12	17		0.959
MVHR (95% CI)[b]	0.75 (0.16 - 3.50)	1.14 (0.51 - 2.56)	Ref.	1.41 (0.62 - 3.19)	1.36 (0.57 - 3.24)	2.02 (0.90 - 4.58)	0.060	
**Females**								
Person-years	13,590	59,570	44,134	62,938	60,409	123,401		
CRC cases	1	4	5	7	9	28		
MVHR (95% CI)[b]	0.64 (0.07 - 5.47)	0.60 (0.16 - 2.23)	Ref.	0.98 (0.31 - 3.08)	1.31 (0.44 - 3.91)	2.00 (0.77 - 5.20)	0.005	
**Diabetes (N=27486)**								
**Total**								
Person-years	10,962	109,586	87,263	72,979	39,438	31,935		
CRC cases	13	196	166	130	61	35		
MVHR (95% CI)[c]	0.64 (0.36 - 1.14)	0.90 (0.73 - 1.11)	Ref.	1.09 (0.86 - 1.38)	1.06 (0.79 - 1.43)	1.14 (0.78 - 1.65)	0.029	
**Males**								
Person-years	5,736	61,017	44,603	36,365	16,621	7,994		
CRC cases	10	128	104	80	35	15		0.861

© 2024 Paragomi P et al. *JAMA Network Open*.

	BMI (kg/m^2)							
	< 18.5	18.5 - 23	23 - 25	25 - 27.5	27.5 - 30	≥ 30	*P for trend*	$P_{heterogeneity}$[i]
MVHR (95% CI)[d]	0.82 (0.42 - 1.58)	0.96 (0.74 - 1.24)	Ref.	1.18 (0.87 - 1.59)	1.18 (0.80 - 1.74)	1.40 (0.80 - 2.42)	0.045	
**Females**								
Person-years	5,227	48,569	42,660	36,615	22,818	23,942		
CRC cases	3	68	62	50	26	20		
MVHR (95% CI)[d]	0.32 (0.09 - 1.08)	0.86 (0.61 - 1.23)	Ref.	0.94 (0.64 - 1.38)	0.91 (0.57 - 1.45)	0.97 (0.58 - 1.62)	0.338	
**No diabetes (N=429779)**								
**Total**								
Person-years	362,727	2,747,538	1,523,170	1,094,430	467,127	305,046		
CRC cases	407	3093	1741	1248	510	250		
MVHR (95% CI)[c]	0.92 (0.83 - 1.03)	0.98 (0.92 - 1.04)	Ref.	1.08 (1.01 - 1.16)	1.20 (1.08 - 1.32)	1.31 (1.14 - 1.50)	<.001	
**Males**								
Person-years	164,963	1,281,975	700,237	502,985	184,988	82,938		
CRC cases	221	1835	986	689	235	101		0.350
MVHR (95% CI)[d]	0.88 (0.75 - 1.01)	0.94 (0.87 - 1.02)	Ref.	1.10 (1.00 - 1.21)	1.18 (1.02 - 1.36)	1.43 (1.16 - 1.76)	<.001	
**Females**								
Person-years	197,764	1,465,563	822,933	591,446	282,139	222,109		
CRC cases	186	1258	755	559	275	149		
MVHR (95% CI)[d]	0.99 (0.84 - 1.17)	1.03 (0.94 - 1.12)	Ref.	1.06 (0.95 - 1.19)	1.21 (1.06 - 1.39)	1.25 (1.05 - 1.50)	0.005	
**Current smokers (N=126703)**								
**Total**								
Person-years	134,929	897,844	424,140	278,423	98,907	40,728		
CRC cases	186	1270	622	388	130	47		
MVHR (95% CI)[e]	0.88 (0.75 - 1.04)	0.90 (0.82 - 1.00)	Ref.	1.07 (0.94 - 1.21)	1.05 (0.87 - 1.27)	1.11 (0.83 - 1.50)	<.001	
**Males**								
Person-years	109,159	789,692	377,308	249,400	85.818	32,021		
CRC cases	153	1141	565	357	114	41		0.374
MVHR (95% CI)[f]	0.88 (0.74 - 1.06)	0.90 (0.81 - 0.99)	Ref.	1.08 (0.94 - 1.23)	1.13 (0.92 - 1.38)	1.22 (0.88 - 1.67)	<.001	
**Females**								
Person-years	25,770	108,152	46,833	29,024	13,089	8,707		
CRC cases	33	129	57	31	16	6		
MVHR (95% CI)[f]	0.87 (0.56 - 1.36)	0.97 (0.70 - 1.34)	Ref.	0.96 (0.62 - 1.50)	0.72 (0.40 - 1.28)	0.70 (0.30 - 1.62)	0.535	
**Former or never smokers (N=335282)**								
**Total**								
Person-years	258,436	2,032,598	1,205,221	900,322	411,809	298,770		
CRC cases	247	2080	1301	1006	443	243		
MVHR (95% CI)[e]	0.92 (0.80 - 1.06)	1.01 (0.94 - 1.08)	Ref.	1.10 (1.02 - 1.20)	1.24 (1.11 - 1.38)	1.37 (1.19 - 1.57)	<.001	
**Males**								
Person-years	63,721	561,454	369,657	291,589	116,225	59,136		
CRC cases	80	836	524	412	158	75		0.318
MVHR (95% CI)[f]	0.84 (0.67 - 1.07)	1.01 (0.90 - 1.13)	Ref.	1.13 (1.00 - 1.29)	1.25 (1.05 - 1.50)	1.60 (1.25 - 2.04)	<.001	
**Females**								
Person-years	194,716	1,471,144	835,565	608,733	295,584	239,635		
CRC cases	167	1244	777	594	285	168		
MVHR (95% CI)[f]	0.98 (0.83 - 1.16)	1.02 (0.93 - 1.11)	Ref.	1.07 (0.96 - 1.20)	1.21 (1.06 - 1.39)	1.28 (1.08 - 1.52)	<.001	
**Ever alcohol users (N=136181)**								
**Total**								
Person-years	109,779	910,583	499,129	345,451	123,258	45,018		
CRC cases	165	1252	768	511	172	76		
MVHR (95% CI)[g]	0.92 (0.78 - 1.09)	0.89 (0.81 - 0.98)	Ref.	1.04 (0.93 - 1.16)	1.08 (0.92 - 1.28)	1.33 (1.05 - 1.68)	<.001	
**Males**								
Person-years	71,641	661,829	385,404	272,163	93,432	30,788		
CRC cases	123	1051	649	443	143	62		0.209
MVHR (95% CI)[h]	0.92 (0.76 - 1.12)	0.89 (0.81 - 0.99)	Ref.	1.09 (0.96 - 1.23)	1.10 (0.92 - 1.32)	1.50 (1.16 - 1.95)	<.001	
**Females**								
Person-years	38,139	248,755	113,725	73,289	29,826	14,231		
CRC cases	42	201	119	68	29	14		
MVHR (95% CI)[h]	0.83 (0.58 - 1.22)	0.86 (0.68 - 1.09)	Ref.	0.79 (0.59 - 1.07)	0.93 (0.62 - 1.41)	0.89 (0.50 - 1.56)	0.816	
**Never alcohol users (N=300333)**								
**Total**								
Person-years	262,354	1,834,609	1,054,852	795,678	376,047	292,590		
CRC cases	245	1829	1044	807	387	206		
MVHR (95% CI)[g]	0.97 (0.84 - 1.11)	1.04 (0.96 - 1.12)	Ref.	1.11 (1.01 - 1.22)	1.26 (1.12 - 1.42)	1.30 (1.11 - 1.51)	<.001	

© 2024 Paragomi P et al. *JAMA Network Open*.

	BMI (kg/m²)							
	**< 18.5**	**18.5 - 23**	**23 - 25**	**25 - 27.5**	**27.5 - 30**	**≥ 30**	***P* for trend**	***P*$_{heterogeneity}$[i]**
**Males**								
Person-years	91,680	599,971	317,907	245,713	102,733	59,026		
CRC cases	95	760	368	278	118	50		0.830
MVHR (95% CI)[h]	0.86 (0.68 - 1.08)	1.02 (0.90 - 1.15)	Ref.	1.10 (0.94 - 1.29)	1.32 (1.07 - 1.63)	1.36 (1.01 - 1.84)	<.001	
**Females**								
Person-years	170,675	1,234,638	736,945	549,965	274,214	233,564		
CRC cases	150	1069	676	529	269	156		
MVHR (95% CI)[h]	1.06 (0.88 - 1.26)	1.06 (0.96 - 1.17)	Ref.	1.11 (0.99 - 1.24)	1.24 (1.07 - 1.43)	1.29 (1.08 - 1.54)	0.008	

BMI: body mass index, CRC: colorectal cancer [a]Model adjusted for age at baseline, sex, education, marital status, diabetes at baseline, current smoking, ever alcohol use, and enrollment period

[b]Model adjusted for age at baseline, education, marital status, diabetes at baseline, current smoking, ever alcohol use, and enrollment period

[c]Model adjusted for age at baseline, sex, education, marital status, current smoking, ever alcohol use, and enrollment period

[d]Model adjusted for age at baseline, education, marital status, current smoking, ever alcohol use, and enrollment period

[e]Model adjusted for age at baseline, sex, education, marital status, diabetes at baseline, ever alcohol use, and enrollment period

[f]Model adjusted for age at baseline, education, marital status, diabetes at baseline, ever alcohol use, and enrollment period

[g]Model adjusted for age at baseline, sex, education, marital status, diabetes at baseline, current smoking, and enrollment period

[h]Model adjusted for age at baseline, education, marital status, diabetes at baseline, current smoking, and enrollment period

[i]P value was obtained form the interaction test between BMI and sex after model adjustment

© 2024 Paragomi P et al. *JAMA Network Open*.

eTable 14. Associations Between BMI and CRC Mortality by Sex in Subgroups

	BMI (kg/m²)							
	< 18.5	18.5 - 23	23 - 25	25 - 27.5	27.5 - 30	≥ 30	*P for trend*	$P_{heterogeneity}$[i]
**Chinese origin (N=246222)**								
**Total**								
Person-years	179,987	1,508,446	952.588	690,113	285,038	133,212		
CRC deaths	116	775	483	339	183	90		
MVHR (95% CI)[a]	1.10 (0.90 - 1.36)	1.02 (0.91 - 1.15)	Ref.	1.01 (0.88 - 1.16)	1.29 (1.08 - 1.53)	1.38 (1.10 - 1.73)	0.029	
**Males**								
Person-years	98,147	736,671	419,030	315,227	110,553	38,456		
CRC deaths	84	517	261	189	76	41		0.228
MVHR (95% CI)[b]	1.17 (0.92 - 1.50)	1.04 (0.90 - 1.21)	Ref.	1.05 (0.87 - 1.27)	1.18 (0.92 - 1.53)	1.94 (1.39 - 2.69)	0.124	
**Females**								
Person-years	81,840	771,776	533,559	374,886	174,485	94,756		
CRC deaths	32	258	222	150	107	49		
MVHR (95% CI)[b]	1.00 (0.69 - 1.45)	1.01 (0.84 - 1.21)	Ref.	0.95 (0.77 - 1.17)	1.33 (1.05 - 1.68)	1.08 (0.79 - 1.47)	0.194	
**South Korea (N=58375)**								
**Total**								
Person-years	20,761	245,236	162,054	134,562	49,841	19,301		
CRC deaths	3	39	14	22	6	1		
MVHR (95% CI)[a]	0.98 (0.28 - 3.46)	1.49 (0.81 - 2.76)	Ref.	1.97 (1.01 - 3.86)	1.38 (0.53 - 3.59)	0.66 (0.09 - 5.00)	0.809	
**Males**								
Person-years	6,950	95,069	82,087	73,489	25,292	7,817		
CRC deaths	2	21	6	12	4	0		0.726
MVHR (95% CI)[b]	1.22 (0.24 - 6.17)	1.63 (1.63 – 1.63)	Ref.	2.89 (2.89 – 2.89)	2.88 (2.88 – 2.88)	N/A	-	
**Females**								
Person-years	13,812	150,168	79,968	61,073	24,549	11,485		
CRC deaths	1	18	8	10	2	1		
MVHR (95% CI)[b]	0.71 (0.09 - 5.72)	1.33 (0.57 - 3.06)	Ref.	1.40 (0.55 - 3.54)	0.63 (0.13 - 2.97)	0.74 (0.09 - 5.96)	0.597	
**Japan (N=295580)**								
**Total**								
Person-years	283,144	2,379,774	1,211,435	803,348	286,465	113,051		
CRC deaths	159	1145	564	337	130	64		
MVHR (95% CI)[a]	0.88 (0.74 - 1.05)	0.96 (0.86 - 1.06)	Ref.	0.92 (0.81 - 1.05)	1.04 (0.86 - 1.26)	1.27 (0.98 - 1.65)	0.074	
**Males**								
Person-years	100,592	1,070,941	605,973	383,182	121,638	39,054		
CRC deaths	73	650	340	186	70	36		0.123
MVHR (95% CI)[b]	0.87 (0.67 - 1.12)	0.93 (0.82 - 1.07)	Ref.	0.92 (0.77 - 1.09)	1.14 (0.88 - 1.48)	1.80 (1.28 - 2.54)	0.008	
**Females**								
Person-years	182,553	1,308,833	605,463	420,166	164,828	73,998		
CRC deaths	86	495	224	151	60	28		
MVHR (95% CI)[b]	0.90 (0.70 - 1.17)	0.99 (0.85 - 1.16)	Ref.	0.94 (0.76 - 1.15)	0.96 (0.72 - 1.28)	0.94 (0.64 - 1.40)	0.933	
**Iran (N=50018)**								
**Total**								
Person-years	27,545	134,045	85,631	113,798	99,638	162,088		
CRC deaths	4	14	10	11	13	28		
MVHR (95% CI)[a]	1.16 (0.36 - 3.73)	0.83 (0.37 - 1.88)	Ref.	0.87 (0.37 - 2.04)	1.26 (0.55 - 2.88)	1.92 (0.92 - 4.02)	0.015	
**Males**								
Person-years	13,877	74,218	41,368	50,671	38,986	38,182		
CRC deaths	1	10	7	9	6	10		0.457
MVHR (95% CI)[b]	0.41 (0.05 - 3.34)	0.78 (0.30 - 2.04)	Ref.	1.07 (0.40 - 2.88)	0.96 (0.32 - 2.88)	1.71 (0.64 - 4.53)	0.068	
**Females**								
Person-years	13,669	59,828	44,263	63,127	60,652	123,907		
CRC deaths	3	4	3	2	7	18		
MVHR (95% CI)[b]	2.95 (0.59 - 14.7)	0.96 (0.21 - 4.28)	Ref.	0.48 (0.08 - 2.86)	1.81 (0.47 - 7.02)	2.35 (0.69 - 8.02)	0.105	
**Diabetes (N=36040)**								
**Total**								
Person-years	14,442	154,199	119,292	99,281	50,659	37,636		
CRC deaths	12	133	88	58	39	17		
MVHR (95% CI)[c]	1.06 (0.58 - 1.95)	1.13 (0.86 - 1.48)	Ref.	0.87 (0.62 - 1.21)	1.26 (0.86 - 1.84)	1.01 (0.59 - 1.72)	0.676	
**Males**								
Person-years	7,827	91,010	66,591	52,710	22,779	10,474		

© 2024 Paragomi P et al. *JAMA Network Open*.

	BMI (kg/m^2)						P for trend	$P_{heterogeneity}$[i]
	< 18.5	18.5 - 23	23 - 25	25 - 27.5	27.5 - 30	≥ 30		
CRC deaths	6	93	49	42	19	10		0.138
MVHR (95% CI)[d]	0.99 (0.42 - 2.32)	1.32 (0.93 - 1.86)	Ref.	1.17 (0.78 - 1.77)	1.27 (0.75 - 2.17)	1.74 (0.87 - 3.43)	0.739	
**Females**								
Person-years	6,615	63,190	52,702	46,571	27,880	27,163		
CRC deaths	6	40	39	16	20	7		
MVHR (95% CI)[d]	1.05 (0.44 - 2.50)	0.88 (0.56 - 1.37)	Ref.	0.48 (0.27 - 0.87)	1.10 (0.64 - 1.90)	0.48 (0.21 - 1.10)	0.168	
**No diabetes (N=604076)**								
**Total**								
Person-years	467,942	3,998,116	2,255,679	1,620,256	662,722	385,271		
CRC deaths	257	1782	966	638	289	165		
MVHR (95% CI)[c]	0.96 (0.84 - 1.11)	0.98 (0.90 - 1.06)	Ref.	0.98 (0.89 - 1.09)	1.17 (1.02 - 1.33)	1.45 (1.23 - 1.71)	<.001	
**Males**								
Person-years	208,824	1,872,611	1,077,005	766,659	272,687	112,633		
CRC deaths	154	1093	564	353	136	77		0.135
MVHR (95% CI)[d]	0.99 (0.83 - 1.19)	0.95 (0.86 - 1.06)	Ref.	0.98 (0.86 - 1.12)	1.16 (0.96 - 1.40)	1.89 (1.48 - 2.40)	<.001	
**Females**								
Person-years	259,118	2,125,505	1,178,675	853,597	390,036	272,639		
CRC deaths	103	689	402	285	153	88		
MVHR (95% CI)[d]	0.93 (0.75 - 1.16)	1.01 (0.89 - 1.14)	Ref.	0.99 (0.85 - 1.15)	1.17 (0.97 - 1.42)	1.20 (0.95 - 1.52)	0.068	
**Current smokers (N=181835)**								
**Total**								
Person-years	169,871	1,298,320	638,399	419,375	147,487	59,066		
CRC deaths	115	781	340	202	71	44		
MVHR (95% CI)[e]	0.95 (0.76 - 1.17)	1.00 (0.88 - 1.13)	Ref.	1.01 (0.85 - 1.20)	1.07 (0.83 - 1.38)	1.77 (1.29 - 2.42)	0.032	
**Males**								
Person-years	137,818	1,147,259	574,904	377,671	128,540	46,689		
CRC deaths	98	698	311	191	63	38		0.062
MVHR (95% CI)[f]	0.94 (0.75 - 1.19)	0.96 (0.84 - 1.10)	Ref.	1.05 (0.87 - 1.25)	1.09 (0.83 - 1.43)	1.96 (1.40 - 2.75)	0.005	
**Females**								
Person-years	32,053	151,062	63,496	41,704	18,948	12,378		
CRC deaths	17	83	29	11	8	6		
MVHR (95% CI)[f]	0.98 (0.53 - 1.79)	1.35 (0.88 - 2.06)	Ref.	0.60 (0.30 - 1.21)	0.92 (0.42 - 2.02)	1.11 (0.46 - 2.68)	0.180	
**Former or never smokers (N=463000)**								
**Total**								
Person-years	332,149	2,926,863	1,755,437	1,311,477	570,031	366,333		
CRC deaths	162	1167	721	502	259	139		
MVHR (95% CI)[e]	0.99 (0.84 - 1.18)	0.99 (0.90 - 1.08)	Ref.	0.97 (0.86 - 1.08)	1.21 (1.05 - 1.40)	1.30 (1.08 - 1.57)	0.004	
**Males**								
Person-years	81,029	824,500	570,837	443,333	167,362	76,644		
CRC deaths	61	498	302	205	93	49		0.392
MVHR (95% CI)[f]	1.06 (0.80 - 1.40)	1.01 (0.87 - 1.17)	Ref.	0.96 (0.81 - 1.15)	1.25 (0.99 - 1.58)	1.79 (1.32 - 2.43)	0.025	
**Females**								
Person-years	251,120	2,102,364	1,184,601	868,145	402,669	289,690		
CRC deaths	101	669	419	297	166	90		
MVHR (95% CI)[f]	0.95 (0.76 - 1.19)	0.97 (0.86 - 1.10)	Ref.	0.96 (0.83 - 1.12)	1.19 (0.99 - 1.42)	1.12 (0.88 - 1.41)	0.065	
**Ever alcohol users (N=198120)**								
**Total**								
Person-years	142,697	1,317,070	733,905	500,945	176,188	64,686		
CRC deaths	94	709	379	219	87	41		
MVHR (95% CI)[g]	0.87 (0.69 - 1.10)	0.93 (0.82 - 1.05)	Ref.	0.92 (0.78 - 1.09)	1.08 (0.85 - 1.36)	1.44 (1.04 - 1.99)	0.027	
**Males**								
Person-years	93,387	956,746	570,135	395,053	133,870	44,685		
CRC deaths	74	585	316	197	74	37		0.005
MVHR (95% CI)[h]	0.93 (0.72 - 1.21)	0.93 (0.81 - 1.06)	Ref.	1.01 (0.84 - 1.20)	1.16 (0.90 - 1.49)	1.84 (1.31 - 2.60)	0.002	
**Females**								
Person-years	49,311	360,324	163,770	105,892	42,318	20,002		
CRC deaths	20	124	63	22	13	4		
MVHR (95% CI)[h]	0.67 (0.40 - 1.11)	0.89 (0.65 - 1.20)	Ref.	0.52 (0.32 - 0.85)	0.77 (0.42 - 1.40)	0.46 (0.17 - 1.25)	0.282	

© 2024 Paragomi P et al. *JAMA Network Open*.

	BMI (kg/m²)							
	**< 18.5**	**18.5 - 23**	**23 - 25**	**25 - 27.5**	**27.5 - 30**	**≥ 30**	***P for trend***	$P_{heterogeneity}$[i]
**Never alcohol users (N=421155)**								
**Total**								
Person-years	337,754	2,721,126	1,583,831	1,191,780	530,718	358,808		
CRC deaths	172	1149	648	467	242	141		
MVHR (95% CI)[g]	1.04 (0.88 - 1.23)	1.03 (0.93 - 1.13)	Ref.	1.01 (0.89 - 1.14)	1.25 (1.08 - 1.45)	1.40 (1.16 - 1.68)	0.003	
**Males**								
Person-years	115,735	924,780	531,625	402,552	156,096	77,287		
CRC deaths	79	549	273	187	81	50		0.336
MVHR (95% CI)[h]	1.08 (0.84 - 1.39)	1.04 (0.90 - 1.20)	Ref.	1.00 (0.83 - 1.20)	1.24 (0.97 - 1.59)	1.95 (1.44 - 2.65)	0.027	
**Females**								
Person-years	222,019	1,796,347	1,052,206	789,228	374,622	281,521		
CRC deaths	93	600	375	280	161	91		
MVHR (95% CI)[h]	1.01 (0.80 - 1.28)	1.02 (0.90 - 1.16)	Ref.	1.01 (0.87 - 1.18)	1.25 (1.04 - 1.51)	1.19 (0.94 - 1.51)	0.067	

BMI: body mass index, CRC: colorectal cancer

[a]Model adjusted for age at baseline, sex, diabetes at baseline, current smoking, and ever alcohol use

[b]Model adjusted for age at baseline, diabetes at baseline, current smoking, and ever alcohol use

[c]Model adjusted for age at baseline, sex, current smoking, and ever alcohol use

[d]Model adjusted for age at baseline, current smoking, and ever alcohol use

[e]Model adjusted for age at baseline, sex, diabetes at baseline, and ever alcohol use

[f]Model adjusted for age at baseline, diabetes at baseline, and ever alcohol use

[g]Model adjusted for age at baseline, sex, diabetes at baseline, and current smoking

[h]Model adjusted for age at baseline, diabetes at baseline, and current smoking

[i]P value was obtained form the interaction test between BMI and sex after model adjustment

© 2024 Paragomi P et al. *JAMA Network Open*.

eTable 15. Associations Between BMI and CRC Mortality by Sex in Subgroups Among Those With Complete Data on Covariates

	BMI (kg/m^2)						*P* for trend	$P_{heterogeneity}$[i]
	< 18.5	18.5 - 23	23 - 25	25 - 27.5	27.5 - 30	≥ 30		
**Chinese origin (N=238097)**								
**Total**								
Person-years	175,315	1,470,627	928,019	669,767	278,027	130,754		
CRC deaths	116	760	473	332	179	88		
MVHR (95% CI)[a]	1.12 (0.91 - 1.38)	1.02 (0.91 - 1.15)	Ref.	1.01 (0.88 - 1.17)	1.28 (1.08 - 1.52)	1.36 (1.08 - 1.71)	0.044	
**Males**								
Person-years	93,494	699,189	394,608	294,986	103,607	35,998		
CRC deaths	84	502	251	182	72	39		0.145
MVHR (95% CI)[b]	1.20 (0.93 - 1.54)	1.04 (0.89 - 1.21)	Ref.	1.06 (0.87 - 1.28)	1.17 (0.90 - 1.52)	1.91 (1.36 - 2.68)	0.177	
**Females**								
Person-years	81,822	771,438	533,412	374,782	174,420	94,756		
CRC deaths	32	258	222	150	107	49		
MVHR (95% CI)[b]	0.99 (0.68 - 1.44)	1.01 (0.84 - 1.21)	Ref.	0.95 (0.77 - 1.18)	1.35 (1.05 - 1.68)	1.07 (0.79 - 1.47)	0.205	
**South Korea (N=58375)**								
**Total**								
Person-years	20,761	245,236	162,054	134,562	49,841	19,301		
CRC deaths	3	39	14	22	6	1		
MVHR (95% CI)[a]	0.95 (0.27 - 3.34)	1.47 (0.79 - 2.72)	Ref.	1.97 (1.01 - 3.85)	1.41 (0.54 - 3.68)	0.66 (0.09 - 5.05)	0.732	
**Males**								
Person-years	6,950	95,069	82,087	73,489	25,292	7,817		
CRC deaths	2	21	6	12	4	0		0.622
MVHR (95% CI)[b]	1.16 (0.23 - 5.89)	1.63 (0.65 - 4.07)	Ref.	2.82 (1.05 - 7.53)	3.00 (0.84 - 10.69)	N/A	0.248	
**Females**								
Person-years	13,812	150,168	79,968	61,073	24,549	11,485		
CRC deaths	1	18	8	10	2	1		
MVHR (95% CI)[b]	0.67 (0.08 - 5.43)	1.29 (0.56 - 2.99)	Ref.	1.43 (0.56 - 3.62)	0.64 (0.14 - 3.03)	0.76 (0.10 - 6.10)	0.688	
**Japan (N=150691)**								
**Total**								
Person-years	195,627	1,306,994	584,735	370,453	131,535	51,703		
CRC deaths	112	667	306	174	63	30		
MVHR (95% CI)[a]	0.77 (0.62 - 0.96)	0.87 (0.76 - 1.00)	Ref.	0.92 (0.76 - 1.11)	1.01 (0.77 - 1.32)	1.12 (0.77 - 1.63)	0.012	
**Males**								
Person-years	67,036	582,204	291,109	175,98-	55,679	17,443		
CRC deaths	49	360	174	95	34	17		0.140
MVHR (95% CI)[b]	0.76 (0.55 - 1.05)	0.85 (0.70 - 1.02)	Ref.	0.97 (0.75 - 1.24)	1.18 (0.82 - 1.71)	1.77 (1.07 - 2.91)	<.001	
**Females**								
Person-years	128,592	724,790	293,626	194,474	75,856	34,261		
CRC deaths	63	307	132	79	29	13		
MVHR (95% CI)[b]	0.78 (0.58 - 1.06)	0.90 (0.74 - 1.11)	Ref.	0.88 (0.66 - 1.16)	0.86 (0.58 - 1.29)	0.77 (0.43 - 1.35)	0.739	
**Iran (N=49903)**								
**Total**								
Person-years	27,479	133,715	85,510	113,603	99,386	161,644		
CRC deaths	4	14	10	11	13	28		
MVHR (95% CI)[a]	1.19 (0.37 - 3.82)	0.85 (0.38 - 1.92)	Ref.	0.86 (0.36 - 2.02)	1.22 (0.53 - 2.80)	1.88 (0.90 - 3.95)	0.022	
**Males**								
Person-years	13,877	74,135	41,355	50,629	38,955	38,141		
CRC deaths	1	10	7	9	6	10		0.349
MVHR (95% CI)[b]	0.43 (0.05 - 3.53)	0.80 (0.30 - 2.10)	Ref.	1.03 (0.38 - 2.78)	0.89 (0.30 - 2.68)	1.61 (0.61 - 4.28)	0.115	
**Females**								
Person-years	13,603	59,580	44,156	62,974	60,431	123,503		
CRC deaths	3	4	3	2	7	18		
MVHR (95% CI)[b]	2.87 (0.58 - 14.3)	0.95 (0.21 - 4.26)	Ref.	0.48 (0.08 - 2.90)	1.83 (0.47 - 7.09)	2.37 (0.69 - 8.10)	0.100	
**Diabetes (N=28339)**								
**Total**								
Person-years	11,060	111,905	90,020	75,775	41,234	32,750		
CRC deaths	10	100	74	40	31	16		
MVHR (95% CI)[c]	1.06 (0.54 - 2.07)	1.06 (0.78 - 1.44)	Ref.	0.70 (0.48 - 1.04)	1.12 (0.73 - 1.71)	0.99 (0.57 - 1.73)	0.484	
**Males**								
Person-years	5,781	62,454	46,024	37,898	17,452	8,397		

© 2024 Paragomi P et al. *JAMA Network Open*.

	BMI (kg/m^2)							
	< 18.5	18.5 - 23	23 - 25	25 - 27.5	27.5 - 30	≥ 30	*P for trend*	$P_{heterogeneity}$[i]
CRC deaths	5	65	39	26	12	9		0.234
MVHR (95% CI)[d]	1.03 (0.40 - 2.65)	1.17 (0.78 - 1.74)	Ref.	0.89 (0.54 - 1.47)	0.93 (0.49 - 1.79)	1.74 (0.83 - 3.65)	0.910	
**Females**								
Person-years	5,28-	49,452	43,996	37,877	23,783	24,354		
CRC deaths	5	35	35	14	19	7		
MVHR (95% CI)[d]	1.06 (0.41 - 2.74)	0.94 (0.58 - 1.50)	Ref.	0.47 (0.25 - 0.88)	1.12 (0.64 - 1.95)	0.47 (0.21 - 1.07)	0.126	
**No diabetes (N=458648)**								
**Total**								
Person-years	379,069	2,929,481	1,633,561	1,190,327	509,953	325,907		
CRC deaths	212	1322	712	486	226	130		
MVHR (95% CI)[c]	0.94 (0.80 - 1.10)	0.95 (0.87 - 1.04)	Ref.	1.02 (0.91 - 1.15)	1.20 (1.03 - 1.40)	1.42 (1.18 - 1.72)	<.001	
**Males**								
Person-years	172,661	1,374,867	758,273	553,985	205,078	90,601		
CRC deaths	131	816	398	271	103	57		0.146
MVHR (95% CI)[d]	0.99 (0.81 - 1.22)	0.94 (0.83 - 1.06)	Ref.	1.07 (0.91 - 1.24)	1.22 (0.98 - 1.51)	1.87 (1.42 - 2.48)	<.001	
**Females**								
Person-years	206,409	1,554,614	875,289	634,343	304,876	235,307		
CRC deaths	81	506	314	215	123	73		
MVHR (95% CI)[d]	0.87 (0.68 - 1.11)	0.97 (0.84 - 1.12)	Ref.	0.97 (0.82 - 1.16)	1.19 (0.96 - 1.47)	1.18 (0.91 - 1.53)	0.026	
**Current smokers (N=134167)**								
**Total**								
Person-years	138,687	945,570	452,056	301,868	108,803	44,821		
CRC deaths	98	580	237	146	52	29		
MVHR (95% CI)[e]	0.96 (0.75 - 1.22)	1.00 (0.86 - 1.17)	Ref.	1.06 (0.86 - 1.30)	1.11 (0.82 - 1.51)	1.60 (1.08 - 2.35)	0.073	
**Males**								
Person-years	113,013	836,510	404,932	272,410	95,647	35,847		
CRC deaths	85	509	215	136	45	26		0.019
MVHR (95% CI)[f]	0.97 (0.75 - 1.25)	0.95 (0.81 - 1.11)	Ref.	1.08 (0.87 - 1.34)	1.10 (0.80 - 1.52)	1.85 (1.23 - 2.79)	0.016	
**Females**								
Person-years	25,675	109,061	47,124	29,459	12,156	8,974		
CRC deaths	13	71	22	10	7	3		
MVHR (95% CI)[f]	0.94 (0.47 - 1.88)	1.62 (1.00 - 2.63)	Ref.	0.79 (0.37 - 1.67)	1.17 (0.50 - 2.75)	0.77 (0.23 - 2.58)	0.194	
**Former or never smokers (N=357539)**								
**Total**								
Person-years	271,078	2,168,685	1,290,392	975,550	446,521	316,328		
CRC deaths	132	875	556	388	207	118		
MVHR (95% CI)[e]	0.95 (0.78 - 1.15)	0.95 (0.85 - 1.05)	Ref.	0.97 (0.85 - 1.11)	1.21 (1.03 - 1.42)	1.30 (1.06 - 1.60)	<.001	
**Males**								
Person-years	67,624	608,949	401,511	321,108	127,318	63,376		
CRC deaths	50	382	222	162	71	40		0.334
MVHR (95% CI)[f]	1.01 (0.74 - 1.37)	0.99 (0.84 - 1.17)	Ref.	1.03 (0.84 - 1.26)	1.25 (0.95 - 1.63)	1.84 (1.31 - 2.59)	0.007	
**Females**								
Person-years	203,454	1,559,736	888,882	654,442	319,204	252,952		
CRC deaths	82	493	334	226	136	78		
MVHR (95% CI)[f]	0.91 (0.71 - 1.17)	0.92 (0.80 - 1.06)	Ref.	0.93 (0.79 - 1.11)	1.18 (0.97 - 1.45)	1.12 (0.87 - 1.44)	0.030	
**Ever alcohol users (N=139622)**								
**Total**								
Person-years	110,764	932,490	513,641	359,016	128,258	47,292		
CRC deaths	78	514	263	158	62	34		
MVHR (95% CI)[g]	0.90 (0.69 - 1.16)	0.92 (0.79 - 1.07)	Ref.	0.94 (0.77 - 1.15)	1.08 (0.82 - 1.42)	1.67 (1.16 - 2.39)	0.019	
**Males**								
Person-years	72,826	683,512	399,536	283,308	98,226	32,907		
CRC deaths	59	421	218	143	53	33		<.001
MVHR (95% CI)[h]	0.93 (0.69 - 1.24)	0.91 (0.77 - 1.08)	Ref.	1.04 (0.84 - 1.29)	1.16 (0.86 - 1.56)	2.30 (1.59 - 3.32)	<.001	
**Females**								
Person-years	37,938	248,979	114,105	73,709	30,033	14,386		
CRC deaths	19	93	45	15	9	1		
MVHR (95% CI)[h]	0.79 (0.46 - 1.37)	0.92 (0.64 - 1.32)	Ref.	0.51 (0.28 - 0.91)	0.78 (0.38 - 1.60)	0.16 (0.02 - 1.19)	0.094	
**Never alcohol users (N=326524)**								
**Total**								
Person-years	277,433	1,994,777	1,152,705	880,274	416,454	311,952		

© 2024 Paragomi P et al. *JAMA Network Open*.

	BMI (kg/m^2)							
	**< 18.5**	**18.5 - 23**	**23 - 25**	**25 - 27.5**	**27.5 - 30**	**≥ 30**	***P* for trend**	***P*$_{heterogeneity}$[i]**
CRC deaths	141	851	496	358	196	112		
MVHR (95% CI)[g]	0.98 (0.81 - 1.19)	0.98 (0.88 - 1.10)	Ref.	1.02 (0.89 - 1.17)	1.28 (1.08 - 1.51)	1.30 (1.06 - 1.61)	0.001	
**Males**								
Person-years	98,086	671,714	362,927	284,811	118,804	64,956		
CRC deaths	70	408	195	143	62	33		0.754
MVHR (95% CI)[h]	1.08 (0.82 - 1.42)	1.00 (0.84 - 1.18)	Ref.	1.06 (0.85 - 1.32)	1.26 (0.95 - 1.69)	1.61 (1.11 - 2.34)	0.051	
**Females**								
Person-years	179,347	1,323,064	789,779	595,463	297,650	246,997		
CRC deaths	71	443	301	215	134	79		
MVHR (95% CI)[h]	0.91 (0.70 - 1.18)	0.98 (0.84 - 1.13)	Ref.	0.99 (0.83 - 1.18)	1.26 (1.03 - 1.55)	1.19 (0.92 - 1.53)	0.017	

BMI: body mass index, CRC: colorectal cancer

[a]Model adjusted for age at baseline, sex, education, marital status, diabetes at baseline, current smoking, ever alcohol use, and enrollment period

[b]Model adjusted for age at baseline, education, marital status, diabetes at baseline, current smoking, ever alcohol use, and enrollment period

[c]Model adjusted for age at baseline, sex, education, marital status, current smoking, ever alcohol use, and enrollment period

[d]Model adjusted for age at baseline, education, marital status, current smoking, ever alcohol use, and enrollment period

[e]Model adjusted for age at baseline, sex, education, marital status, diabetes at baseline, ever alcohol use, and enrollment period

[f]Model adjusted for age at baseline, education, marital status, diabetes at baseline, ever alcohol use, and enrollment period

[g]Model adjusted for age at baseline, sex, education, marital status, diabetes at baseline, current smoking, and enrollment period

[h]Model adjusted for age at baseline, education, marital status, diabetes at baseline, current smoking, and enrollment period

[i]P value was obtained form the interaction test between BMI and sex after model adjustment

© 2024 Paragomi P et al. *JAMA Network Open*.

eTable 16. Associations Between BMI (Actual Measurement) and CRC Incidence by Sex

	BMI (kg/m²)						P for trend	P for interaction[c]
	< 18.5	18.5 - 23	23 - 25	25 - 27.5	27.5 - 30	≥ 30		
**All subjects**								
**Total**								
Person-years	94,057	1,003,480	660,512	564,377	229,422	101,358		
CRC cases	52	658	485	512	251	100		
MVHR (95% CI)[a]	0.76 (0.57 - 1.02)	0.96 (0.85 - 1.08)	Ref.	1.16 (1.02 - 1.31)	1.29 (1.11 - 1.51)	1.12 (0.90 - 1.39)	<.001	
**Males**								
Person-years	36,864	362,182	272,466	238,079	82,533	25,962		
CRC cases	22	260	215	211	101	28		0.161
MVHR (95% CI)[b]	0.70 (0.45 - 1.09)	0.87 (0.72 - 1.04)	Ref.	1.13 (0.94 - 1.37)	1.49 (1.18 - 1.89)	1.34 (0.90 - 1.98)	<.001	
**Females**								
Person-years	57,194	641,298	388,046	326,299	146,890	75,397		
CRC cases	30	398	270	301	150	72		
MVHR (95% CI)[b]	0.81 (0.56 - 1.19)	1.02 (0.87 - 1.19)	Ref.	1.18 (1.00 - 1.39)	1.20 (0.98 - 1.47)	1.08 (0.83 - 1.40)	0.023	

BMI: body mass index, CRC: colorectal cancer
[a]Model adjusted for age at baseline, sex, diabetes at baseline, current smoking, ever alcohol use, and enrollment period
[b]Model adjusted for age at baseline, diabetes at baseline, current smoking, ever alcohol use, and enrollment period
[c]P value was obtained form the interaction test between BMI and sex after model adjustment

eTable 17. Associations Between BMI (Self-Report) and CRC Incidence by Sex

	BMI (kg/m²)						P for trend	P for interaction[c]
	< 18.5	18.5 - 23	23 - 25	25 - 27.5	27.5 - 30	≥ 30		
**All subjects**								
**Total**								
Person-years	230,774	2,252,526	1,315,250	820,799	297,380	118,358		
CRC cases	343	3500	2158	1442	543	241		
MVHR (95% CI)[a]	0.91 (0.81 - 1.02)	0.96 (0.91 - 1.02)	Ref.	1.07 (1.00 - 1.15)	1.15 (1.04 - 1.26)	1.34 (1.18 - 1.54)	<.001	
**Males**								
Person-years	114,802	1,179,003	678,175	426,415	137,905	45,408		
CRC cases	210	2260	1366	914	310	123		0.775
MVHR (95% CI)[b]	0.91 (0.79 - 1.06)	0.95 (0.89 - 1.02)	Ref.	1.08 (1.00 - 1.18)	1.15 (1.02 - 1.30)	1.43 (1.19 - 1.72)	<.001	
**Females**								
Person-years	115,972	1,073,524	637,075	394,384	159,476	72,951		
CRC cases	133	1240	792	528	233	118		
MVHR (95% CI)[b]	0.90 (0.75 - 1.09)	0.99 (0.90 - 1.08)	Ref.	1.06 (0.95 - 1.18)	1.15 (0.99 - 1.33)	1.28 (1.05 - 1.55)	<.001	

BMI: body mass index, CRC: colorectal cancer
[a]Model adjusted for age at baseline, sex, diabetes at baseline, current smoking, ever alcohol use, and enrollment period
[b]Model adjusted for age at baseline, diabetes at baseline, current smoking, ever alcohol use, and enrollment period
[c]P value was obtained form the interaction test between BMI and sex after model adjustment

© 2024 Paragomi P et al. *JAMA Network Open*.

eTable 18. Associations Between BMI (Actual Measurement) and CRC Mortality by Sex

	BMI (kg/m²)						P for trend	P for interaction[c]
	< 18.5	18.5 - 23	23 - 25	25 - 27.5	27.5 - 30	≥ 30		
**All subjects**								
**Total**								
Person-years	109,109	1,173,580	766,608	658,143	272,395	122,303		
CRC deaths	36	339	232	235	137	58		
MVHR (95% CI)[a]	1.10 (0.77 - 1.56)	1.05 (0.89 - 1.24)	Ref.	1.07 (0.90 - 1.29)	1.37 (1.11 - 1.70)	1.25 (0.93 - 1.67)	0.042	
**Males**								
Person-years	42,827	443,098	325,974	286,121	102,141	33,649		
CRC deaths	17	171	97	102	50	19		0.412
MVHR (95% CI)[b]	1.19 (0.71 - 2.00)	1.23 (0.96 - 1.58)	Ref.	1.19 (0.90 - 1.58)	1.54 (1.09 - 2.16)	1.83 (1.12 - 2.99)	0.157	
**Females**								
Person-years	66,282	730,483	440,634	372,022	170,255	88,655		
CRC deaths	19	168	135	133	87	39		
MVHR (95% CI)[b]	1.03 (0.64 - 1.67)	0.91 (0.73 - 1.14)	Ref.	0.99 (0.78 - 1.26)	1.26 (0.96 - 1.65)	1.03 (0.72 - 1.47)	0.117	

BMI: body mass index, CRC: colorectal cancer
[a]Model adjusted for age at baseline, sex, diabetes at baseline, current smoking, ever alcohol use, and enrollment period
[b]Model adjusted for age at baseline, diabetes at baseline, current smoking, ever alcohol use, and enrollment period
[c]P value was obtained form the interaction test between BMI and sex after model adjustment

© 2024 Paragomi P et al. *JAMA Network Open*.

eTable 19. Associations Between BMI (Self-Report) and CRC Mortality by Sex

	BMI (kg/m²)						P for trend	P for interaction[c]
	**< 18.5**	**18.5 - 23**	**23 - 25**	**25 - 27.5**	**27.5 - 30**	**≥ 30**		
**All subjects**								
**Total**								
Person-years	239,843	2,354,904	1,373,479	861,572	312,246	123,830		
CRC deaths	160	1242	697	391	155	88		
MVHR (95% CI)[a]	1.02 (0.86 - 1.21)	1.00 (0.91 - 1.10)	Ref.	0.95 (0.84 - 1.08)	1.08 (0.91 - 1.29)	1.59 (1.27 - 1.99)	0.079	
**Males**								
Person-years	119,328	1,233,540	712,144	449,536	145,237	47,739		
CRC deaths	111	840	457	255	88	51		0.228
MVHR (95% CI)[b]	1.06 (0.86 - 1.31)	0.96 (0.86 - 1.08)	Ref.	0.94 (0.81 - 1.10)	1.04 (0.83 - 1.31)	1.88 (1.40 - 2.51)	0.083	
**Females**								
Person-years	120,515	1,121,364	661,335	412,037	167,010	76,092		
CRC deaths	49	402	240	136	67	37		
MVHR (95% CI)[b]	0.94 (0.69 - 1.28)	1.10 (0.93 - 1.29)	Ref.	0.97 (0.78 - 1.20)	1.16 (0.88 - 1.52)	1.35 (0.95 - 1.91)	0.495	

BMI: body mass index, CRC: colorectal cancer
[a]Model adjusted for age at baseline, sex, diabetes at baseline, current smoking, ever alcohol use, and enrollment period
[b]Model adjusted for age at baseline, diabetes at baseline, current smoking, ever alcohol use, and enrollment period
[c]P value was obtained form the interaction test between BMI and sex after model adjustment

© 2024 Paragomi P et al. *JAMA Network Open*.
