# Supplementary material for: Body Mass Index and Risk of Colorectal Cancer Incidence and Mortality in Asia
Source: JAMA Netw Open. 2024 Aug 28;7(8):e2429494. doi: 10.1001/jamanetworkopen.2024.29494 (PMC11358861; doi:10.1001/jamanetworkopen.2024.29494)
Supplement: Supplement 2. — Data Sharing Statement [file jamanetwopen-e2429494-s002.pdf]

Data Sharing Statement

Paragomi. Body Mass Index and Risk of Colorectal Cancer Incidence and Mortality in Asia. *JAMA Netw Open*. Published August 28, 2024. doi:10.1001/jamanetworkopen.2024.29494

Data
Data available: No

Additional Information
Explanation for why data not available: The data underlying this manuscript were obtained from the Asia Cohort Consortium (ACC) and cannot be shared publicly due to the privacy of study participants. Researchers can apply for access to these data through the National Cancer Centre Japan after obtaining ethical approval from an Institutional Review Board.
